# Novel insights into electrical double layers in carbonate reservoirs under low-salinity water injection using molecular dynamics simulation

**DOI:** 10.1038/s41598-025-14647-z

**Published:** 2025-08-23

**Authors:** Saifali Al-Musawi, Fariborz Rashidi, Sepideh Amjad-Iranagh

**Affiliations:** 1 Department of Petroleum Engineering, Amirkabir University of Technology, (Tehran Polytechnic), Tehran, Iran; 2 Department of Chemical Engineering, Amirkabir University of Technology, (Tehran Polytechnic), Tehran, Iran; 3 Department of Materials and Metallurgical Engineering, Amirkabir University of Technology, Tehran Polytechnic, Tehran, Iran

**Keywords:** Rock-brine interactions, Low salinity water injection, Porous medium simulation, Carbonate reservoir rock, Electrical double layer, Molecular dynamics simulation, Petrology, Geochemistry

## Abstract

Understanding how mineral heterogeneity and brine salinity influence the electrical double layer (EDL) structure is crucial for optimizing low-salinity water injection strategies in carbonate reservoirs. In this study, molecular dynamics (MD) simulations were conducted to elucidate the mechanisms governing EDL behavior at the interface of a novel composite carbonate surface (87% calcite, 13% quartz) in contact with diluted water (DW), seawater (SW), and high-salinity formation water (FW) in presence of complex model oil including wide variety of nonpolar oil hydrocarbon. Results indicated that increasing brine salinity compressed the Stern layer significantly, strengthening electrostatic interactions at the mineral surface and promoting oil adhesion due to enhanced ion bridging by divalent cations (Ca²⁺, Mg²⁺). This process resulted in a pronounced shift toward hydrophobic (oil-wet) surface characteristics under FW conditions, compared to SW and DW. Additionally, the observed differences in water–surface interactions between calcite and quartz were explained mechanistically: the highly charged calcite surface enabled strong inner-sphere coordination of water molecules at 2.37 Å, whereas the neutral quartz surface supported only weaker outer-sphere hydration at 3.1 Å. Ion mobility analysis further revealed enhanced dynamics of Na⁺ ions in SW system, facilitating disruption of oil–surface ionic bridges, while FW promoted ion clustering and reduced ionic mobility, stabilizing oil attachment. These insights emphasize the critical role of mineral surface chemistry and salinity-dependent electrostatic interactions in governing wettability and oil recovery efficiency during low-salinity waterflooding.

**Supplementary Information:**

The online version contains supplementary material available at 10.1038/s41598-025-14647-z.

## Introduction

It is well-known that Low Salinity Water Injection (LSWI) into a reservoir disturbance the system’s equilibrium involving rock, brine, and oil. Numerous physicochemical interactions lead to increasing the oil recovery due to the new equilibrium in the system. These interactions are thought to provide the foundation for the interpretation of LSWI mechanisms in both sandstone and carbonate reservoirs. They may have occurred at the fluid/fluid (between oil and brine) interface, the fluid/rock (between brine and rock) interface, or both. Previous studies have generally proposed a wide range of mechanisms^1–3^. It is established that wettability alteration is one of the main mechanisms enhancing oil recovery (EOR) upon LSWI. While the exact mechanisms remain a topic of ongoing research and debate, various explanations have been proposed to support the alteration of reservoir rock wettability. These include migration of fine particles^4,5^ multi-ion exchange (MIE)^6,7^ expansion of EDL^8–10^ hydration effect^11^ pH effect^12^ and salt in and salt out effect^13^.

Oil reservoirs are classified into various rock types, including sandstones, limestones, dolomites, conglomerates, shales, igneous rocks, and metamorphic rocks. Sandstones and carbonate rocks are the predominant reservoir rocks, accounting for 30% and 60% of global oil and gas production, respectively^14^. Understanding the depositional environment and natural phenomena that influence these attributes is crucial for analyzing petroleum reservoirs. LSWI applies to carbonate and sedimentary reservoirs, and its performance and governing mechanisms are significantly influenced by all rock compositions^15^. The mechanism of oil recovery from carbonate and sandstone reservoirs is influenced by the chemical compositions of minerals under varying salinities and ionic compositions^16,17^. The adsorption or desorption of hydrocarbon components is influenced by the composition of carbonate rocks, which range from neutral to relatively positive in charge. The varying wetness of each rock surface also significantly impacts the mechanism’s efficacy. Calcite, the most abundant mineral, plays a crucial role in soil formation in oil reservoirs, and surface features of calcite are essential for oil recovery efficiency^18^. The expansion of the EDL is a key factor in alterations in wettability, with studies showing that tuning the electrolyte content in reinjected water during water flooding increases oil and gas recovery^19^. However, many carbonate reservoirs also contain quartz (SiO), the second most abundant mineral on Earth. Quartz is commonly found in carbonate reservoirs alongside calcite and other phases^20^. Despite this, its role in Low Salinity Water Injection (LSWI) processes has received limited attention. The behavior of the EDL varies notably between calcite and quartz due to differences in mineralogy and surface charge, which may alter brine–rock interactions. Understanding the role of such heterogeneities is essential for optimizing LSWI performance in realistic reservoir settings^10,21–23^.

Calcite (CaCO₃) is the principal mineral in carbonate reservoirs, where low-salinity waterflooding (LSW) has emerged as a promising EOR technique due to its potential for wettability alteration. Molecular dynamics (MD) simulations are widely employed to investigate the nanoscale mechanisms behind LSW, often using the calcite (101̅4) surface as the model substrate. This specific plane is chosen because it is the most stable and naturally exposed cleavage surface of calcite under reservoir conditions^24,25^. Its inherent charge neutrality and ability to form structured hydration layers make it ideal for simulating realistic oil–brine–rock interactions relevant to LSW-induced wettability changes. In a follow-up study, Koleini et al.^24^ examined the ionic structure of the brine film using formation water (~ 215,000 ppm) and synthetic seawater (~ 60,000 ppm) with decane + decanoic acid as oil. They confirmed that high salinity leads to ionic cross-linking between oil and mineral, while low salinity supports EDLs and a more water-wet condition. useing fully atomistic MD simulations to study calcite/carbonated brine/oil interfaces using decane + benzoic acid as oil, with brines containing Na⁺, Cl⁻, Mg²⁺, SO₄²⁻, and dissolved CO₂. They tested multiple brine recipes (including carbonated seawater with varying Mg²⁺ and SO₄²⁻ concentrations) and showed that divalent ions, especially in the presence of CO₂, promote desorption of polar oil molecules, rearrange interfacial ion layers, and reduce oil viscosity ^25^. Low salinity brine disrupts the equilibrium of the crude oil–brine–rock system, causing monovalent ions (e.g., Na⁺) to replace divalent cations (e.g., Ca²⁺, Mg²⁺) that bridge oil to the rock surface. This ion exchange weakens oil–rock adhesion and promotes a more water-wet condition^6^. The “salt-in/salt-out” mechanism suggests that changes in brine salinity affect the solubility and dispersion of oil components. At low salinity, the formation of water-in-oil micro-dispersions can enhance oil recovery, although in some cases, increased interfacial tension at high pH may reduce recovery efficiency^13^.

Cation hydration significantly impacts the wettability of calcite and quartz, critical for EOR. For calcite, Jiménez-Ángeles and Firoozabadi^26^ and Zhang et al.^27^ showed that divalent cations like Ca²⁺ and Mg²⁺ form strong hydration shells, reducing oil adhesion and enhancing water-wetness. In quartz, Jiménez-Ángeles & Firoozabadi^26^ found that higher salinity reduces hydration layers, increasing oil-surface interactions. However, Yi et al.^28^ and demonstrated that cations with high hydration enthalpy, such as Mg²⁺ and Ca²⁺, strengthen hydrogen bonds and enhance water adsorption under low-salinity conditions, promoting water-wetness. Underwood et al.^29^ provied that in the presence of calcite, divalent cations such as Ca²⁺ in the surrounding brine may promote oil-wetness by forming cation bridges between surface sites and adsorbed organic molecules, whereas monovalent cations like Na⁺ tend to preserve water-wet conditions by enhancing electrostatic repulsion. Injection of low salinity water may mobilize fine particles from the rock surface, potentially altering the oil–water interface and aiding oil displacement^4^. LSWF can increase the brine pH via ion exchange at the mineral surface. This may lead to deprotonation of acidic groups in crude oil, affecting interfacial tension (IFT) and wettability. However, the extent of pH change and its impact vary across studies^12^.

MD studies show that quartz (001) surfaces, particularly the O-middle terminations, undergo dissociative water adsorption, forming Si–OH groups that significantly influence surface charge and wettability^30^. These alterations affect the EDL structure and ion interactions, which are key mechanisms in wettability alteration and enhanced oil recovery. In brine, positively charged calcite surfaces facilitate the development of a structured EDL by attracting and binding anions from the surrounding solution^31^ Henderson, E emphasized the role of salinity and ionic composition in shaping EDL thickness and adsorption behavior^31^. Anvari and Choi reported that lower salinity expands the EDL, decreasing oil adhesion via enhanced electrostatic repulsion^32^. Conversely Gu et al. showed that divalent cations, like Ca²⁺, compress the EDL, strengthening ionic interactions and influencing wettability^15^. Quartz surfaces with a predominantly negative charge exhibit distinct EDL properties. Low salinity conditions, as shown by Tian and Wang^33^ and Tian et al.^34^ promote EDL expansion and brine film thickening, enhancing hydrophilicity. Conversely, higher salinity compresses the EDL, complicating oil detachment Fang et al.^35^. Sun et al.^36^ demonstrated that divalent cations, such as Ca²⁺, further compress the EDL on quartz, enabling cation-bridging effects that enhance oil-wetness. For quartz, MIE significantly impacts surface wettability through cation-specific interactions. Tian et al.^37^ found that Ca²⁺ forms stable oil-wet bridges across all concentrations, whereas K⁺ bridging is limited to medium ionic strengths. Diluting brine and removing divalent cations, particularly Ca²⁺enhances quartz hydrophilicity by reinforcing EDL repulsion and breaking cation bridges. Sun et al. demonstrated that Ca²⁺ in water films increases hydrophobicity via cation bridging, while Na⁺ enhances water-wetness, highlighting the importance of ion composition^36^. These findings collectively underscore the importance of precise control over brine chemistry to optimize wettability and improve oil recovery in quartz and calcite systems.

However, earlier molecular dynamics simulations of LSWF had some basic issues that needed to be covered. Issues include: (1) use of complicated rock surface. There has been no simulation on this topic. (2) Accurate thermodynamic conditions that account for underground porous medium temperature and pressure. (3) Combining oil compounds. The modeling of all oil components -heavy and light- was not found. This research aims to construct a molecular dynamics model for a real carbonate reservoir sample to gain a thorough understanding of the wettability alterations phenomena. The model will focus on the EDL between the carbonate rock composites and the porous media fluid in the EOR mechanism.

## Materials and simulation method

The composition of the carbonate reservoir rock was determined using XRF and XRD analyses, as summarized in Table 1, additional information can be found in table.S3 and figure.S1 of the Supplementary Information. The results indicate that calcite (87%) and quartz (13%) are the dominant mineral phases in the rock. Although trace amounts of other minerals may exist in natural carbonates, they were excluded from the model due to their negligible abundance and the computational limitations of molecular dynamics simulations. This simplified mineralogy allows for a clearer investigation of the core mechanisms governing electrical double layer (EDL) formation and wettability alteration—two key objectives of this study.

**Fig. 1:**
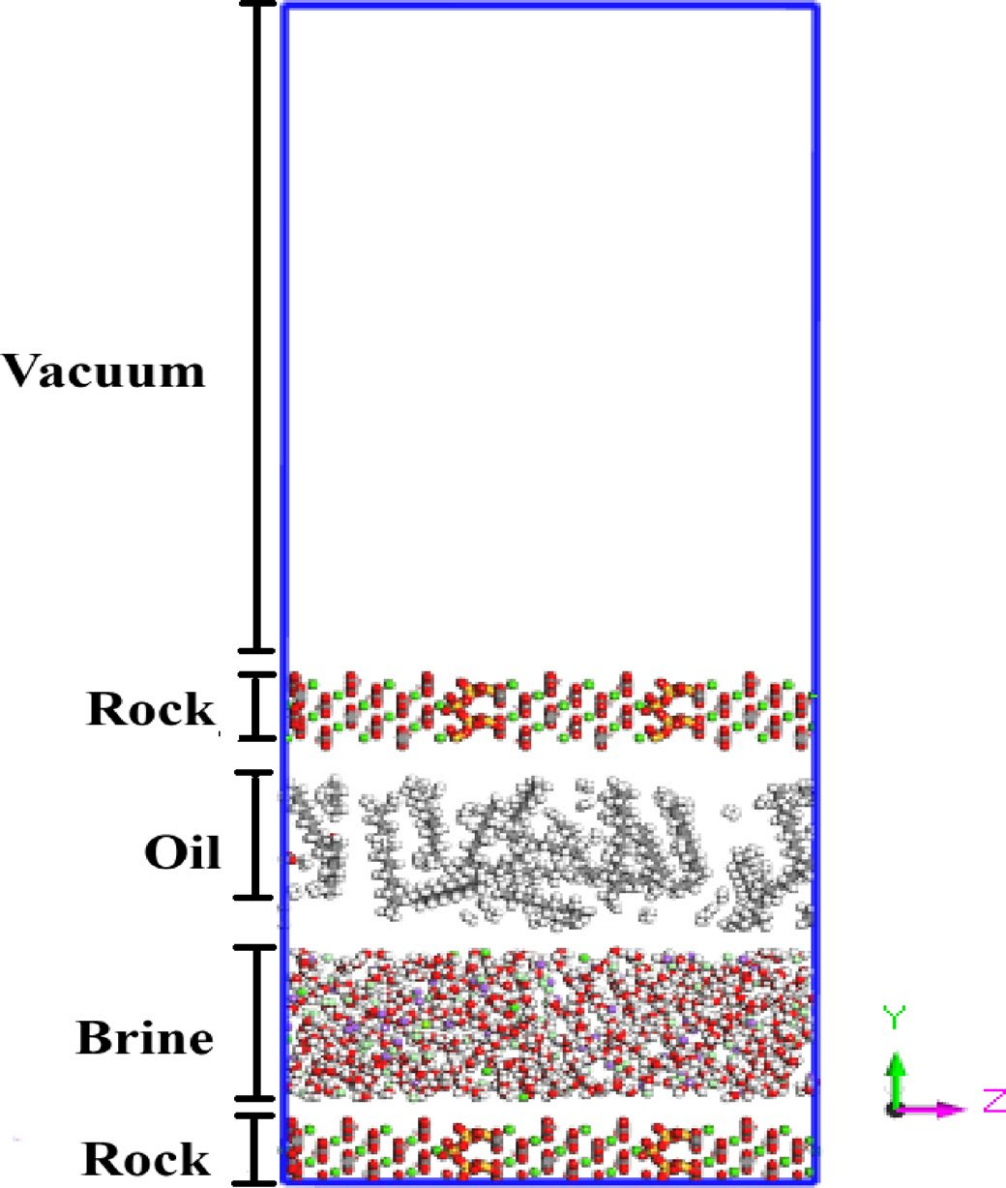
The ultimate design of the system after combining the various components of the simulation system at 0 timesteps (before equilibration).

The heterogeneous solid surface was constructed using Materials Studio software, based on the mineral proportions in Table 1 and as illustrated in Fig. 1. A multi-step modeling protocol was followed.

1. **Individual optimization**: The unit cells of calcite (CaCO₃) and quartz (SiO₂) were separately relaxed using energy minimization to obtain equilibrium geometries.
2. **Supercell construction**: Supercells were generated for both minerals to achieve lateral lattice compatibility and minimize interfacial mismatch.
3. **Interface assembly**: The cleaved (10̅10) surface of calcite (in its hexagonal description^38,39^ and the (001) surface of quartz were aligned to form a planar interface orthogonal to the y-axis.
4. **Energy minimization**: The composite slab was further minimized to remove any residual stress or strain at the interface.

The final composite surface had dimensions of 21.05 × 9.8 × 60.8 Å and included 720 CaCO₃ and 136 SiO₂ atoms. The quartz surface was modeled in its non-hydroxylated form, dominated by siloxane (Si–O–Si) bridges. This weakly polar surface exhibits limited interaction with water, consistent with the absence of a distinct hydration peak, as observed in prior studies^30,40^.

Periodic boundary conditions were applied in all three spatial directions to preserve structural continuity and eliminate artificial edge effects. To prevent interactions between periodic images along the surface normal, an 80 Å vacuum layer was introduced above the upper rock surface. The entire system underwent structural relaxation to eliminate residual stresses, ensuring stability of the calcite–quartz interface prior to introducing fluid phases. This composite surface was subsequently used as the solid substrate in brine interaction simulations.

**Table 1:** Percentage of each reported tissue of XRD analysis after correction by XRF analysis for the real core.

Phases	Percentage
CaCO_3_ (calcite)	87%
SiO_2_ (quartz)	13%

In order to accurately simulate the oil fluid, the oil compound testing is based on the data presented in Table S1, and a detailed breakdown of the oil components model are provided in Table S2 of the Supplementary Information. It is important to note that the oil composition percentages were determined for the oil fluid in a single-phase liquid state, under the conditions of a temperature of 295 K and a reservoir pressure of 420 atm.

The key consideration in this study is that the bubble point of the reservoir occurs at a pressure of 332 atm. Based on the properties of the oil fluid in the reservoir under investigation, the fluid remains in a single-phase liquid state only at temperatures and pressures exceeding the bubble point. Therefore, maintaining precise control of both temperature and pressure was one of the most critical challenges throughout the simulation process. The initial configuration of the system, which includes the setup of the simulation environment and the fluid model, is shown in Fig. 1.

To investigate the effect of salinity and ionic composition on oil–rock interfacial behavior, four brine systems with increasing ionic strength were designed: diluted water (DW), seawater (SW), and formation water (FW). This selection enables a stepwise evaluation of salinity effects relevant to injection and in-situ conditions in carbonate reservoirs. As shown in Table 2, the brines differ in the concentration of Na⁺, Cl⁻, Ca²⁺, and Mg²⁺ ions, reflecting their increasing chemical complexity. The simulation slit was initially filled with water molecules at a density of ~ 1 g/cm³ using VMD software, after which salinity levels were adjusted by replacing water molecules with the target number of ions. Water was modeled using the TIP3P flexible model^41^. This design allows identification of trends in electric double layer formation, wettability alteration, and ionic bridging behavior across realistic salinity conditions, thus providing mechanistic insight relevant to LSWI strategies. The interatomic potentials developed by Xiao, Edwards, and Gräter^42^ were employed to accurately represent the structural and interaction characteristics of calcite. The force field used in this study was selected for its proven accuracy in modeling calcite–aqueous interfaces, a key requirement for simulating the EDL in LSWI systems. Its reliability has been confirmed through validation against experimental data, offering an effective balance between computational efficiency and predictive accuracy. Due to its extensive application in previous studies, it also ensures reproducibility and consistency with existing literature. These interatomic potentials, which have been widely employed in simulations involving calcite and organic compounds^19,43–45^ are summarized in Table S5 of the Supplementary Information. While the quartz rock surface and the oil molecules were modeled using the Consistent Valence Force Field (CVFF).To constrain the water molecules, the SHAKE algorithm was applied. Ions were added to each system according to the compositions outlined in Table 2, enabling the simulation of the respective salinity layers. An example of the final brine configuration is presented in Fig. 1.

Our study utilized a predetermined number of water molecules based on experimental and computational constraints, providing a practical framework for examining interaction mechanisms. This approach allowed for DLVO-type interpretations for films of specific thicknesses but limited the range of disjoining pressures explored. Future research could address this limitation by dynamically adjusting water molecules during simulations better to capture transitions between thin and moderate film thicknesses. Integrating atomic-scale simulations with DLVO-based models could enhance understanding of disjoining pressure, particularly for thin films where classical DLVO theory is inadequate. Experimental validation of these findings would further advance insights into interfacial phenomena across diverse conditions.

**Table 2:** Salinities are based on the number of atoms in each Brine component.

Abbreviated brine identity	Na^+^	Cl^−^	Mg^2+^	Ca^2+^	Water	Oil
DW	15	15	0	0	648	913
SW	50	72	2	9	543	913
FW	65	99	3	14	495	913

In summary, due to the complexity of molecular dynamics simulations and the distinct capabilities of each software, a combination of tools was employed to enhance simulation accuracy. VMD was used to prepare brine solutions with specified ion concentrations. Materials Studio facilitated the construction of composite rock and oil models with varied compositions, atomic charge assignment, and force field coupling. Finally, LAMMPS integrated these components to perform molecular dynamics simulations under controlled temperature and pressure conditions. This multi-software approach is a common practice to leverage the strengths of each program and ensure reliable and precise simulation results.

## Evaluation of simulations

To confirm the credibility of the research acquired, the proposed system should be assessed in conjunction with certain laboratory data to ensure that the simulation performed was accurate. Therefore, two different methods of evaluating the system have been proposed in this research:

1. Viscosity evaluation: The viscosity of the oil system is used to evaluate the oil system, for further details, please refer to Table S4 and Fig. S2 in the Supplementary Information.
2. Contact angle measurement: Evaluations of the system’s surface are carried out by estimating the contact angle between the rock and the fluid. The mechanism is described in the Supplementary Information section of System Evaluation^39,43^. More information can be found in Figs. S3, S4 and S5 of the Supplementary Information.

The simulations were carried out using the LAMMPS^46^ software with a 1 fs simulation timestep. Van der Waals interactions were shortened at a distance cut-off of 12 Å. Beyond 12 Å in reciprocal space, long-range Coulomb interactions were resolved with an accuracy of $\:{10}^{-5}$ using the particle − particle − particle − mesh (PPPM) approach^47^. With the Nose Hoover thermostat connected to the fluid particles and the temperature of the fluid set to 420.0 K^47,48^ all simulations were conducted while the box volume was held constant. To establish equilibrium, the simulation was run for 0.5 ns. During this period, the atoms in the bottom rock slab were frozen in position to maintain their stability, while the top slab was treated as a rigid body, behaving as a single cohesive unit. Since the upper slab was not attached to anything, it was free to rise and fall as the contained brine expanded and contracted according to the thermostat setting of 420.0 K, relevant data are illustrated in Figs. S6 and S7 of the Supplementary Information. In this way, the top slab rises and falls between the vacuum layer 80 Å and the oil slab acting like a solid free piston. It is important to note that the volume change of the contained brine is made possible by the upward/downward movement of the top slab, which is made possible by the existence of a vacuum layer 1 nm thick above the bottom composite rock carbonate surface as shown in many studies such as^24,49^. At the same temperature, a force equivalent to a pressure of 420.0 atm was applied to the upper slab for the subsequent 1 ns. The rock slab functions as a piston, pressing the oil and brine in the reservoir to the ideal level. After this step was complete, the upper slab was fixed (similar to the lower one) and the production run began^22^. The simulations used in the production run lasted 20 ns, and the trajectories of the atoms were printed down at 0.5 ps intervals for subsequent examination. A schematic overview of these steps is presented in Fig. S8 of the Supplementary Information.

## Results

To elucidate the role of LSWI in altering interfacial properties, this study focuses on the behavior of the EDL at the surface of composite carbonate reservoir rocks composed of both calcite and quartz. Using MD simulations, we systematically investigated how key brine ions (Na⁺, Cl⁻, Mg²⁺, and Ca²⁺) interact with the heterogeneous surface under varying salinity conditions. The mineralogical contrast between calcite and quartz provides a unique framework to assess ion-specific preferences and localized EDL formation. Our results highlight that salinity not only affects the overall EDL thickness but also modulates the spatial distribution of ions at the distinct calcite–quartz interfaces. These observations reveal critical insights into how composite rock–brine interactions contribute to the effectiveness of the LSWI mechanism, as detailed in the following section.

**Table 3:** Energy interactions between rock surface and various components (H-water, O-water, oil, Na^+^, Cl^–^) in three different systems, all interaction energies are reported in (kcal/mol).

	Water	H-water	O-water	Oil	Na^+^	Cl^−^	∑Na + Cl
*DW*							
CaCO_**3**_	– 3562.6	– 7656.8	4094.2	– 0.70	– 185.56	658.94	
SiO₂	– 602.2	4112.4	– 4714.7	0.19	– 80.69	– 182.89	
CaCO_3_ + SiO₂	**– 4164.9**	**– 3544.4**	**– 620.5**	**– 0.50**	**– 266.25**	**476.04**	**209.7**
*SW*							
CaCO_**3**_	– 1661.6	– 7488.2	5826.6	– 0.81	– 789.60	1479.95	
SiO₂	– 320.0	3859.2	– 4179.2	0.02	374.06	– 627.27	
CaCO_3_ + SiO₂	**– 1981.6**	**– 3629.0**	**1647.4**	**– 0.78**	**– 415.53**	**852.68**	**437.1**
*FW*							
CaCO_**3**_	– 2387.9	– 4392.0	2004.0	– 0.96	– 1593.63	2627.25	
SiO₂	– 285.2	3488.7	– 3773.9	0.13	208.07	– 476.99	
CaCO_3_ + SiO₂	**– 2673.219**	**– 903.3**	**– 1769.8**	**– 0.82**	**– 1385.55**	**2150.25**	**764.7**

Significant values are in bold.

Table 3 presents the energy interactions between components (H-water,  O-water, oil, Na^+^, Cl^−^) in three different systems (DW, SW, and FW) for two rock composite tissues, CaCO_3_ and SiO_2_. The negative values indicate attractive forces, while positive values indicate repulsive forces. The values highlighted in blue in Table 3 are obtained by summing the values of the two rows directly above them.

These values reflect the cumulative interaction energy across all pairs within the specified groups. The unusually high values arise due to the large number of particle pairs contributing to the total energy. The reported values represent the average of the total cumulative energy across all such pairs during production simulation time. This approach accounts for temporal fluctuations and provides a representative value for the interaction energy.

### Interaction between Na^+^ and the rock surface

Table 3 details notable observations regarding the negative interaction between Na + and CaCO_3_ (values of – 185 kcal/mol for DW, – 789 kcal/mol for SW, and − 1539 kcal/mol for FW). As salinity increases, so does the adsorption capacity of CaCO_3_ rock; this suggests that CaCO_3_ rock has an increased capacity for Na^+^ adsorption^50^, which indicates a favorable interaction of Na + ions with CaCO_3_ tissue.

Na + exhibits an initial negative interaction (value ​​of – 80 kcal/mol), expressed by attraction to the SiO_2_ surface. However, as salinity increases, this effect becomes positive (values ​​of 374 kcal/mol for SW and 208 kcal/mol for FW) due to the occupation of the oxygen atoms of the SiO_2_ rock surface with Na^+^ ions in the SW system. indicating that Na^+^ repulsively affects the SiO_2_ surface and causes it to retreat from the surface. This is an unfavorable interaction for the EOR mechanism for two reasons: first, it increases the thickness of the EDL; second, it destroys the integrity of the stern layer that previously appeared on the surface of pure calcite, which was representative of the reservoir rock^9,15,50,51^. In turn, the instability of the stern layer leads to a decrease in EOR.

Nevertheless, when considering the overall interaction of Na⁺ with the entire composite rock surface, the net interaction becomes increasingly negative with salinity. This suggests that the overall force exerted by Na^+^ is attractive to the entire surface of the reservoir rock, indicating a favorable interaction for the EOR mechanism.

### Interaction between Cl^-^ and the rock surface

Another significant observation in Table 3 is the interaction between Cl⁻ ions and CaCO₃ surfaces. Cl⁻ exhibits a repulsive interaction with calcite that becomes increasingly pronounced with rising salinity, as reflected by interaction energies of + 658 kcal/mol, + 1479 kcal/mol, and + 2627 kcal/mol for the IW, SW, and FW systems, respectively. These increasingly positive values indicate that Cl⁻ ions are positioned farther away from the calcite surface at higher salinities. This behavior is further supported by the ion number density profiles shown in Fig. 3, which demonstrate that Cl⁻ distributions become more spatially localized under high-salinity conditions. The greater separation between Cl⁻ and the calcite surface reduces the hydrophilicity of the CaCO₃ phase, thereby negatively affecting key mechanisms related to EOR. This interpretation is consistent with the findings of Dastjerdi et al.^51^. In contrast, Cl^−^ and SiO_2_ show a negative interaction at the beginning of the DW system, wherein Cl^−^ is adsorbed to the SiO_2_ rock. The interaction energy increases to − 667 kcal/mol in the SW system as salinity rises, whereas it decreases to − 476 kcal/mol in FW. This suggests that Cl⁻ ions cease to interact significantly with the SiO₂ surface beyond a certain concentration. In the FW system, this behavior is attributed to the saturation of available surface sites, where Cl⁻ ions occupy all accessible Si sites on the SiO₂ surface. Briefly, it is significant to note that for all three systems under study, the total interaction between Cl^−^ and the reservoir rock surface is positive (repulsion from the rock surface). On the other hand, Cl^−^ prefers to adsorb to the SiO_2_ part of the reservoir rock surface. However, the increase in the Cl^−^ is one of the main factors in creating the difference in electrical charge and, as a result, reduces the hydrophilicity of the rock surface, which leads to a decrease in EOR, Which is also consistent with Ding et al.^52^ observations.

We analyzed the electrostatic interactions of Na⁺ and Cl⁻ ions with the rock surface to quantify the charge-dependent interaction disparity. A stronger attractive force was observed between Na⁺ ions and the negatively charged rock surface, leading to a net positive interaction energy difference favoring Na⁺ adsorption. Meanwhile, the rock surface with Cl^−^ ions exhibits a negative electrostatic force. The difference in electrostatic force values determines the interaction strength of the entire reservoir rock. The discrepancy in this electric charge provides a force-type value on the entire reservoir rock surface. In the ninth row (∑Na^+^+Cl^−^) of Table 3, the interaction rates between Na^+^ and Cl^−^ are listed in Table 3. The interaction energy value ranges from 209 kcal/mol in low-salinity to 764 kcal/mol. The FW system has recorded the most significant increase, leading to the highest reduction in the thickness of the EDL layer. At high salinities, the increasing electric charge difference force between brine ions is the primary factor influencing the reduction in EDL layer thickness. To validate this assertion, the findings derived from molecular dynamics were utilized. The charge density, density profile, and radial distribution function (RDF) plots are presented.

**Fig. 2:**
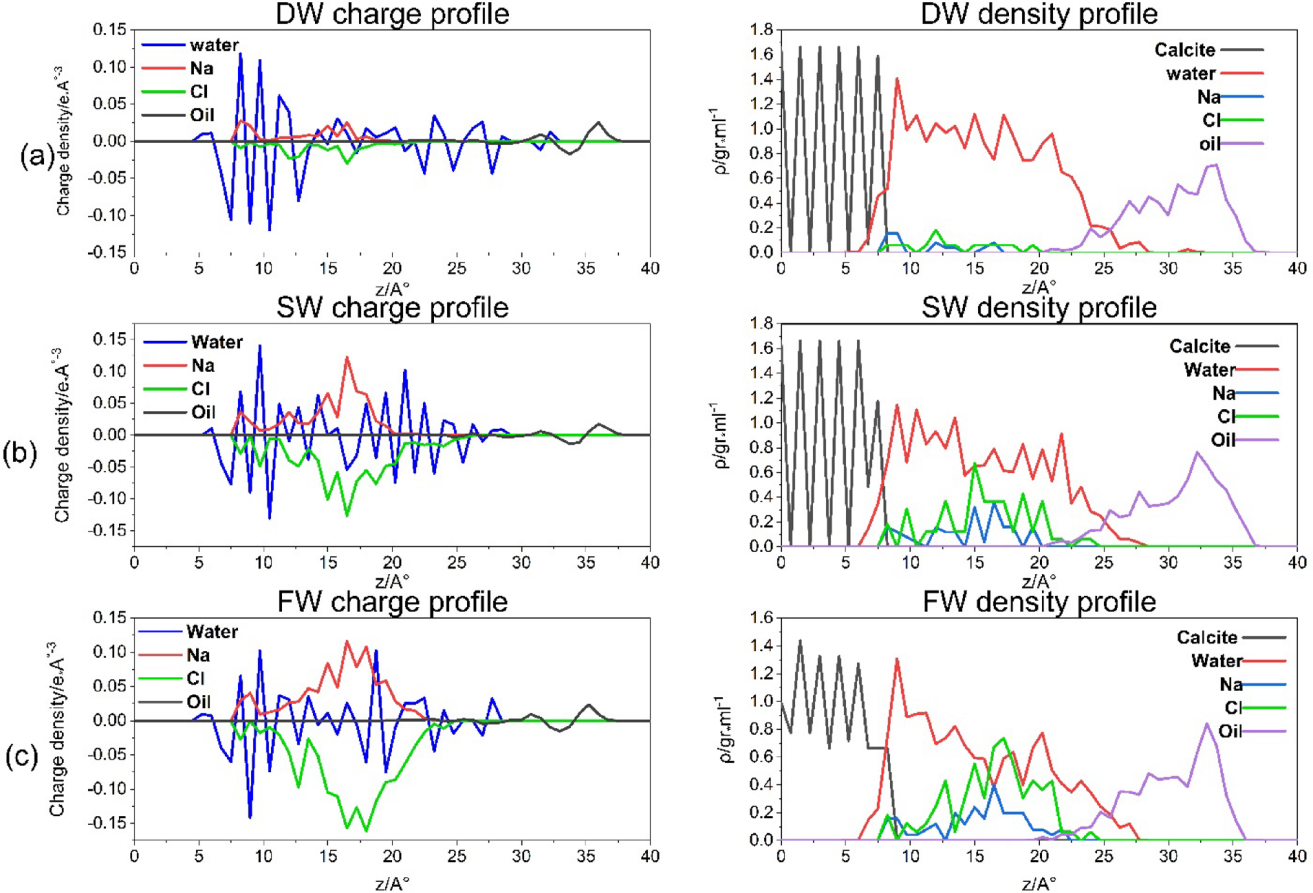
The charge density is on the left side, and the density profile is on the right for DW, SW, and FW systems from top to bottom.

(1) The charge density and density profile:

Figure 2 displays the density diagrams for DW, SW, and FW systems on the right-hand side, while their charge diagram is on the left. The figure displays the salinity increase from top to bottom. purple and yellow lines indicate an increase in both value and area under the curve, which represents the increase in the electric force difference between Na^+^-Cl^−^. This information is also presented in Table 3. It is quantified by numbers. However, the density graphs on the right-hand side of Fig. 2 illustrate the increase in density of Na^+^ and Cl^−^ ions (represented by the blue and green lines) as salinity increases. This change in density is believed to be the primary factor influencing the alteration of surface wettability, which is in agreement with the study^24^. Based on the findings, it can be inferred that the development of the EDL on a charged surface is influenced by the salinity and ionic compositions. Injecting low-ionic strength LSW decreases the repulsive electrostatic forces between the interfaces of oil-brine and brine-rock. This leads to the expansion of the EDL, resulting in the thickening of a thin water layer that separates oil and rock. As a consequence, the wettability of the system shifts towards a water-wet state, facilitating the separation of oil and increasing the oil’s relative permeability.

### The expansion of EDL in the rock surface

Rock surfaces that come into contact with water attract positively charged cations, which are typically hydrated with water molecules, called the Stern layer, and it is devoid of anions. The positive charge of this layer can be caused by two possible causes. First, while the calcite plane is a neutral surface, the outermost atoms of the surface are sets of oxygen atoms of carbonate groups, resulting in relative polarity of the surface with a partial negative charge. Second, polar water molecules create a monolayer on the surface, increasing its polarity. The slab’s improved polarity with partial negative charge due to oxygen atoms makes it a favorable substrate for Na^+^ ions adsorption. Beyond the positive Stern layer, adjacent to the Stern layer is the Diffuse layer, which comprises liberated ions and has a greater concentration of counter ions. The term “EDL” denotes these two layers, and its thickness has been believed to be a significant factor in the mechanism by which wettability changes^53^.

Ion number-density profiles as a function of distance from a cleaved calcite–quartz composite for (A) SW and (B) FW. Gray shading denotes the atomic planes of the rock surfaces; the green region marks the Stern layer (SL), where counterions are tightly adsorbed, and the pink region indicates the diffuse layer (DL), where ion densities decay into the bulk.

**Fig. 3:**
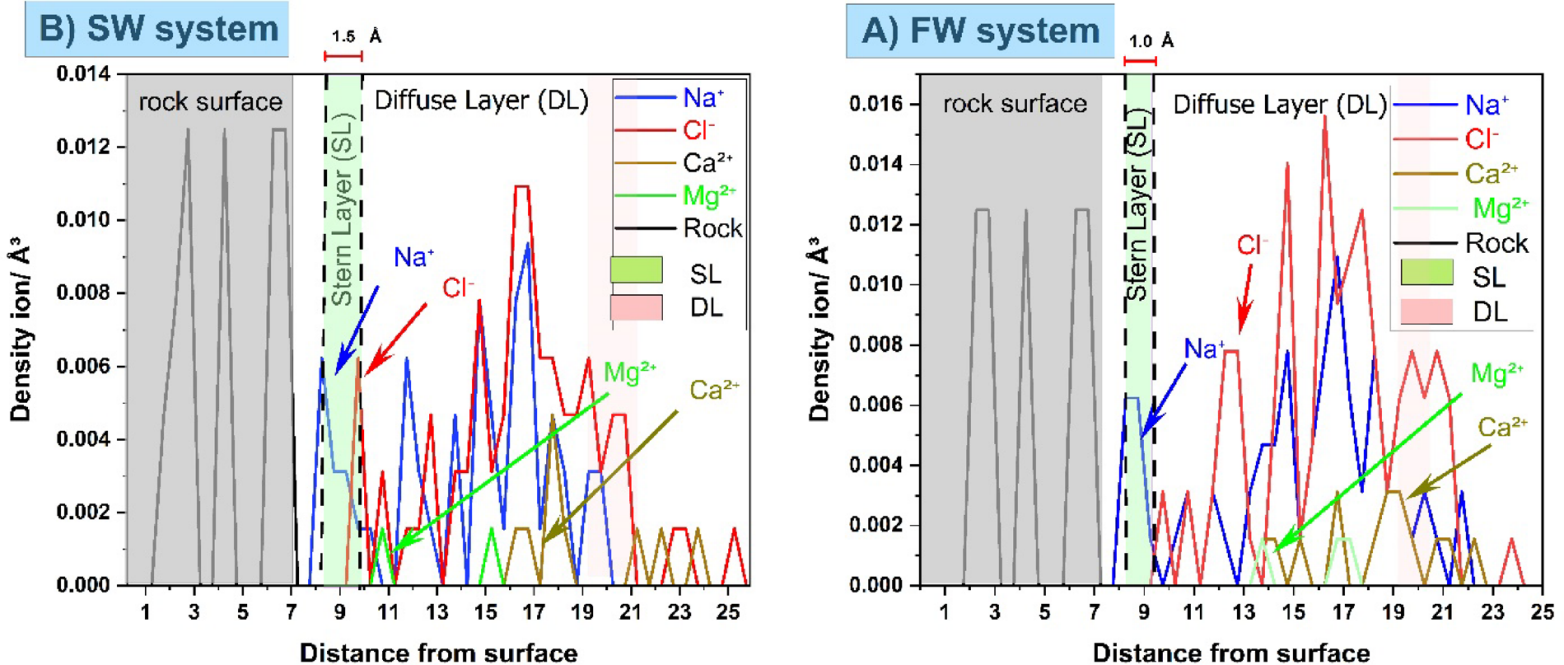
Ion number density profiles for Na⁺, Cl⁻, Ca²⁺, and Mg²⁺, illustrating the formation of EDL on a composite calcite–quartz surface for (**a**) SW and (**b**) FW systems.

**Fig. 4:**
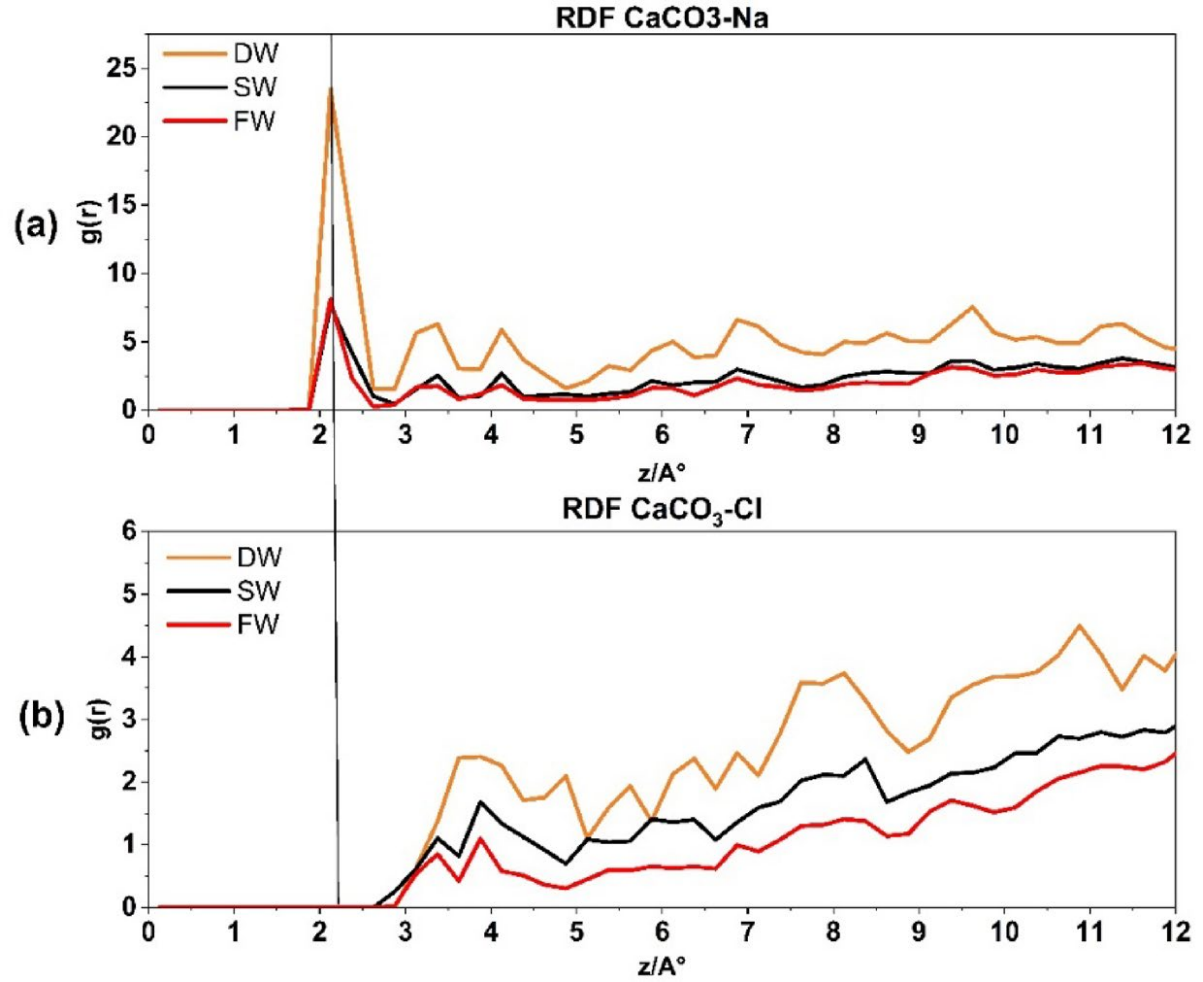
Radial distribution function for CaC**O**_**3**_-**Na**^+^ interaction (**a**) and CaC**O**_**3**_-**Cl**^−^ interaction (**b**).

In SW (Fig. 3A), the SL thickness is approximately 1.5 Å, with Na⁺ and Cl⁻ peaks that are relatively broad and a modest contribution of Ca²⁺/Mg²⁺ reflecting lower ionic strength (interaction energy ≈ 437 kcal/mol). In FW (Fig. 3B), the SL compresses to ~ 1.0 Å, dominated by sharply defined Na⁺ and Cl⁻ peaks (interaction energy ≈ 765 kcal/mol), in agreement with observations by Bourg and Sposito^54^. while divalent cations (Ca²⁺/Mg²⁺) remain less abundant near the surface due to competition and their stronger hydration, specifically for Mg^2+^ ions which exhibit a greater separation from the quartz surface compared to the calcite surface, indicating weaker direct surface interactions with quartz. This behavior is discussed in detail in Fig. 4. Beyond the SL, the first Cl⁻ peak shifts from ~ 10 Å in SW to ~ 12 Å in FW, and both Na⁺ and Cl⁻ distributions become more localized under high salinity. These results confirm that elevating brine salinity compacts the Stern layer and intensifies interfacial electrostatic interactions, thereby promoting ion bridging that enhances oil adhesion and drives the system toward oil-wet conditions, This finding aligns with the results reported by Gu et al.^15^ and Tian et al.^33^.

Figure 4a illustrates the RDF of Na^+^ ions interacting with oxygen on the surface of the CaCO_3_ reservoir rock. The RDF is plotted using a cut-off radius of 12 Å. The RDF is calculated separately for DW, SW, and FW. This allows for analyzing and studying the probable phenomena occurring near the rock within the specified distance. The initial peak is observed at a radius of 2.125 Å, indicating the beginning of Na^+^ ions pairing. The corresponding values are (23, 7, 6). This phrase describes the initial and immediate interaction between Na^+^ ions and the surface of CaCO_3_. The rise in salinity has resulted in a decrease in this correlation, primarily caused by the heightened electric charge difference force between Na^+^ and Cl^−^. This, in turn, leads to a reduction in the thickness of the EDL layer and subsequently decreases the quantity of Na^+^ molecules in the stern layer. Another contributing cause to the peak reduction in this picture is the development of ion bridges due to increased salinity. This phenomenon has been demonstrated in previous study^55^.

In contrast, Fig. 4b illustrates the correlation between Cl^−^ atoms and the CaCO_3_ surface, with DW, SW, and FW. Notably, the first peak appears at a radius of 3.875 Å, indicating that the pairing with calcite commences at a distance of 1.75 Å from the Na^+^ ions (Stern layer). Due to the reasons stated previously, an increase in salinity has an inverse relationship with the number of Cl^−^ molecules that tend to pair with calcite.

Figure 4 presents the RDFs of CaC**O₃**–**Na**^+^, CaC**O₃–Cl**^−^ for different brine systems. This figure provides insights into the affinity of Na⁺, Cl⁻ions for interacting with the calcite surface in the simulated reservoir environment. As expected, Na⁺ ions, which are typically hydrated by water molecules, initially associate with oxygen atoms on the CaCO₃ surface. With increasing salinity, the number of Na⁺ ion pairs decreases, leading to a higher electrostatic force disparity between Na⁺ and Cl⁻ ions. Consequently, this results in a decrease in the thickness of the electrical double layer (EDL), as demonstrated in Fig. 2a–c by the rising density profiles and surface charge densities of Na⁺ lines and Cl⁻ lines across the three systems: DW (A), SW (B), and FW (C).

The presence of Na^+^ and Cl^−^ ions arranged in a layered structure can be noticed on the surface of calcite in brines, as shown in Figs. 4 and 6. The Na^+^ RDF peak adjacent to calcite, within the first compact hydration layer indicates a solid-like form. This shows that the cation is strongly bound directly to the substrate without any water molecules in between. It is important to note that Na^+^ ions are more likely to be adsorbed onto the outermost oxygen of the calcite tissue. At the same time, water molecules are more likely to be adsorbed above the calcium of calcite rock. The AFM experiment conducted by Ricci et al.^56^ provides evidence for the preferential localization of Na^+^ cations on a calcite surface when in contact with an electrolyte solution. The presence of dangling oxygen of calcite atoms, specifically located above the calcium of calcite atoms in the calcite tissue, is responsible for the gap observed between the first peak of water molecules and the peak corresponding to Na^+^ ions near the calcite tissue as it is shown in Fig. 4a.

**Fig. 5:**
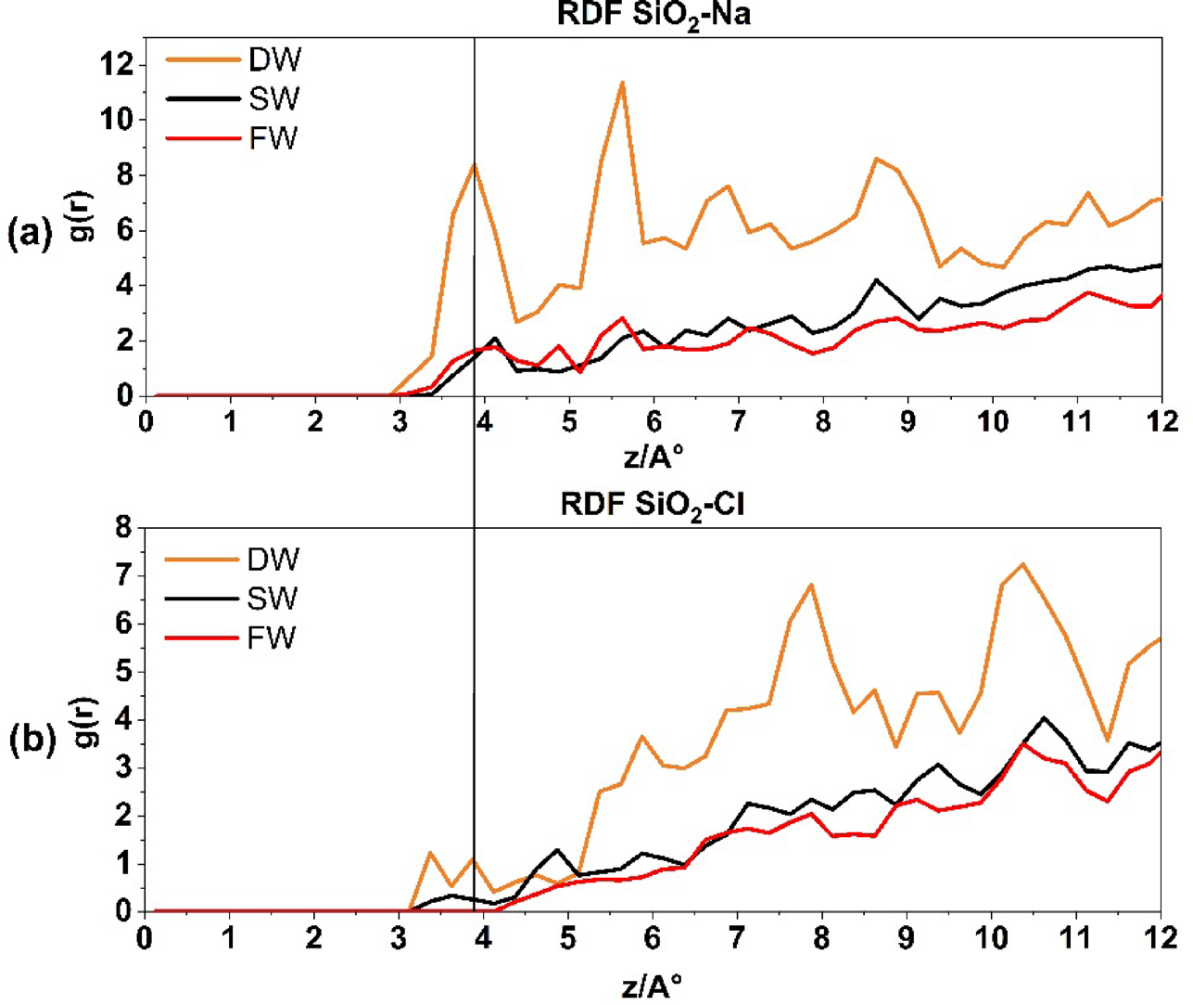
The radial distribution function for Si**O**_**2**_**-Na**^**+**^ interaction in above and Si**O**_**2**_**-Cl**^**−**^ interaction at the down.

Figure 5 depicts the presence of Na^+^ and Cl^−^ ions on the quartz surface. Figure 5a depicts the RDF of Na^+^ ions interacting with the quartz surface of the reservoir rock under different salinity conditions: DW, SW, and FW salinity. In the DW system, the first peak measurement at a radius of 3.875 Å yields a count of 8, suggesting a significant occurrence of Na-quartz rock pairings. As salinity increases, the affinity of Na^+^ to bond with quartz diminishes. However, Fig. 5b illustrates the bonding between Cl^−^ ions and the quartz surface. This bonding is shown by the first peak at a radius of 3.375 Å, which corresponds to a value of 1.2. Conversely, the Na^+^ of Cl^−^ ions exhibits no inclination to establish a connection with quartz rock at lower radii, but its concentration rises at larger radii. It is important to observe that as salinity increases, the propensity of Cl^−^ to interact with SiO_2_ rock diminishes.

**Fig. 6:**
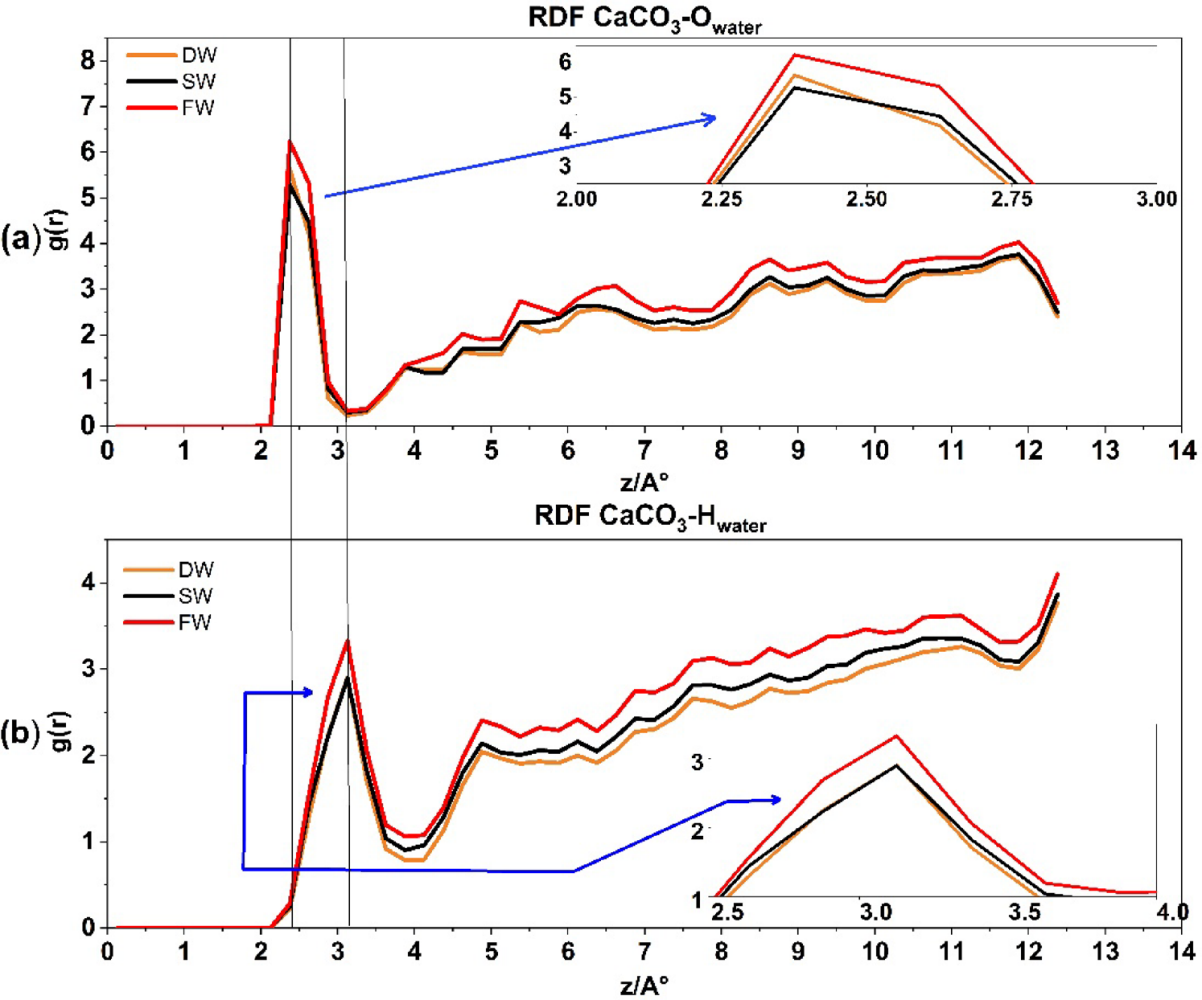
The radial distribution function for **Ca**CO_3_-**O** water interaction in above and **Ca**CO_3_-**H** water interaction at the down.

Figure 6a displays the RDF **Ca**CO_3_-**O**_water_ for all three states of the system. It indicates that as salinity increases, there is a higher likelihood of water oxygen linking with calcite rock (shown by the first peak at $\:\approx\:$ 2.37 Å in Fig. 6a), with a recorded value of $\:\approx\:$ 6.25. The direction of water molecules towards calcite rock is mostly influenced by the oxygen atoms in the water. As salinity increases, the quantity of ions in the porous medium also increases. This causes the EDL to become thinner, resulting in a greater amount of water oxygen molecules that are inclined to bond with calcite. in agreement with recent experimental and simulation results^55^.

The RDF **Ca**CO_3_-**O**–**H** of water in Fig. 6b show that the calcite surface is hydrated by a well-organized layer of water molecules. The density peaks at distances of $\:\approx\:$ 2.3 and 3.1 Å from the surface, which are marked by two horizontal black lines along Fig. 6. These consist of a highly condensed, solid-like layer of water that directly covers the calcite tissue, followed by a less dense and slightly less organized layer of hydration in all levels of salinity. In all salinities, the densities and thicknesses of these hydration layers are nearly identical. The monolayer of water directly above the calcite is almost solid, supporting the mineral’s continued wetness^57^. The X-ray reflectivity results published by Fenter et al.^55^ are consistent with the formation of two water mono-layers on a calcite surface. Furthermore, it was recently proven that calcite’s highly ordered crystal structure initiates the creation of these well-structured hydration layers^58^.

Figure 6b depicts the pairing between H_water_ atoms and Ca_calcite_ rock. The initial peak indicates a 50% decrease ($\:\text{g}\left(\text{r}\right)\approx\:$ 3.12) in the H_water_ atoms pairing with Ca_calcite_ rock compared to O_water_ atoms ($\:\text{g}\left(\text{r}\right)\approx\:$ 6.25) in Fig. 6a. Furthermore, it signifies that the peak of water H_water_ atoms is present at a greater distance, i.e. 3.2 Å, which supports the findings of Ghatee and Koleini 2017 study^59^ and indicates that water is adsorbed to the outermost calcium atoms of calcite through the oxygen atoms.

**Fig. 7:**
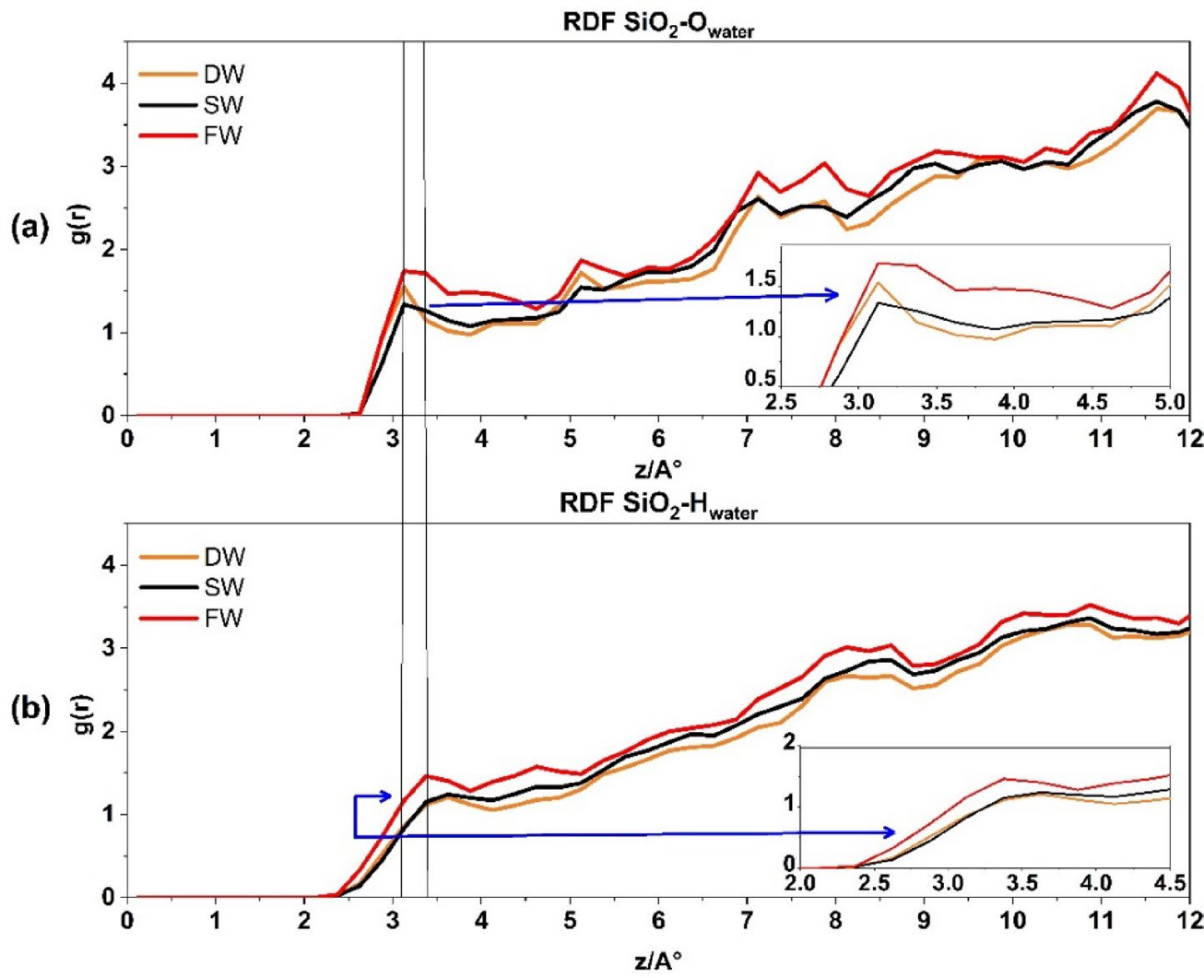
The radial distribution function for **Si**O_2__**O**_water_ interaction in above (**a**) and **Si**O_2__**H**_water_ interaction at the down (**b**).

Figure 7a represents the RDF of SiO2-O_water_ for three systems. The first peak, recorded at g(r) ≈ 1.5, is situated at a distance of ≈ 3.1 Å. The increase in salinity encourages atom pairing with quartz rock. However, Fig. 7b displays the RDF SiO2-H water, which produces the first peak at a greater distance, i.e. 3.3, suggesting the connection of quartz rock and water from the oxygen edge. Previous investigation^36^ support these findings.

A distinct variation has been seen when comparing the interaction between water’s hydrogen and oxygen atoms in quartz and calcite. Water molecules (oxygen and hydrogen) exhibit a comparable process of pairing with quartz rock. Regarding the orientation of water molecules (oxygen and hydrogen), the orientation with quartz rock seems nearly identical. However, in contrast to calcite, quartz does not have a clearly defined peak at the beginning of the graph like calcite. This is a significant difference between the two rock composite tissues, which proves that there is no indication or propensity for a hydrated layer to form on the quartz surface. Conversely, Fig. 6 displays the calcite surface, which indicates the propensity to form hydration layers in all salinities.

**Fig. 8:**
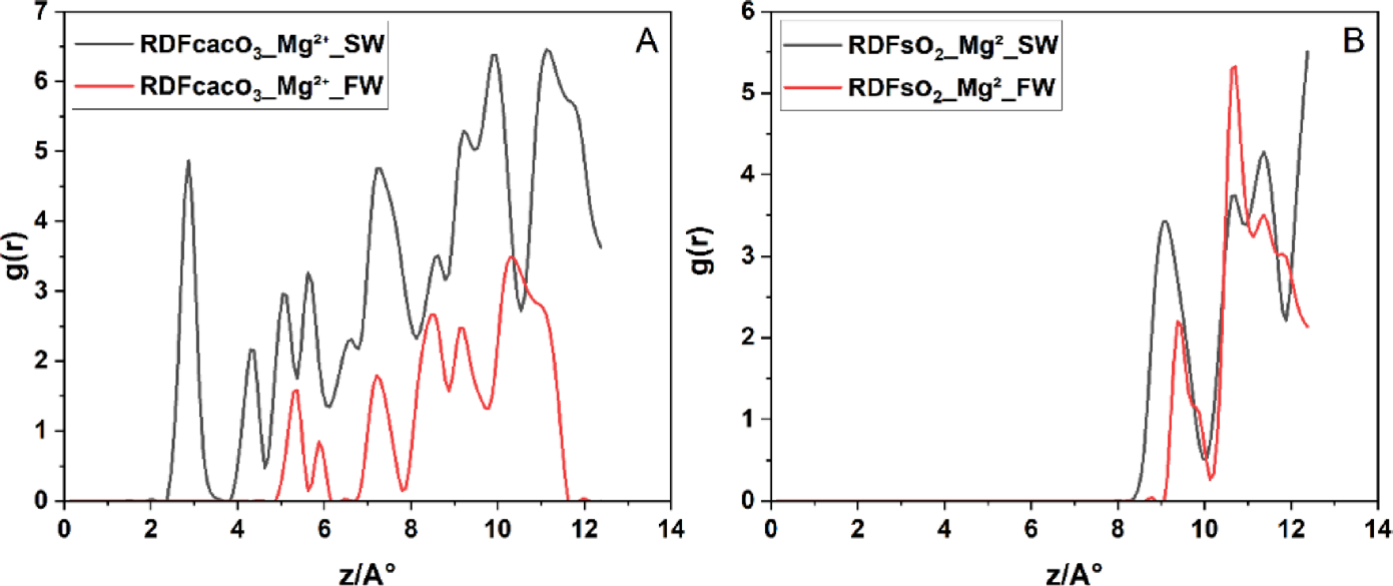
Radial distribution functions (g(r)) of Mg²⁺ relative to surface oxygen atoms on (**A**) calcite surface and (**B**) quartz surface under (SW, black) and (FW, red) systems.

In Fig. 8A, the RDF of CaC**o**_3_-**Mg**^2+^ in SW exhibits a strong inner-sphere peak at ≈ 2,8 Å (g(r) ≈ 4.8), followed by well‐defined second and third hydration‐shell peaks at ≈ 4 Å and ≈ 5 Å. This first‐shell distance and magnitude agree closely with MD reports of direct Mg²⁺ coordination to CaC**o**_3_ surface^60^. Under FW conditions RDF of CaC**o**_3_-**Mg**^2+^ the inner‐sphere peak shifts outward to ≈ 5 Å with markedly lower intensity (g(r) ≈ 1.5), indicating that elevated Ca²⁺ and Na⁺ concentrations compete for adsorption sites and promote Mg²⁺ outer‐sphere, water‐mediated binding^60^. In Fig. 8B, Mg²⁺ in RDF of SiO_2_-**Mg**^2+^ interacts far more weakly with quartz: the primary g(r) peak appears at ≈ 9 Å in SW and shifts slightly to ≈ 10 Å in FW, with peak heights of ~ 3.5 SW and ~ 2.2 FW, confirm that increased ionic strength in FW significantly screens electrostatic interactions, thereby reducing the affinity of Mg²⁺ for the hydrophilic SiO_2_ surface.

Hence, in composite calcite–quartz systems, Mg²⁺ exhibits a salinity-dependent, ion‐specific adsorption behavior: under SW, Mg²⁺ preferentially forms inner‐sphere complexes at calcite’s specific binding sites, while on quartz it remains entirely in outer‐sphere hydration shells. However, in FW, elevated ionic strength and competitive adsorption by monovalent ions disrupt Mg²⁺ inner‐sphere coordination on calcite, shifting it into outer‐sphere configurations on both minerals. This dual influence of mineral heterogeneity and ionic competition critically shapes the electric double layer in carbonate reservoir rocks.

**Fig. 9:**
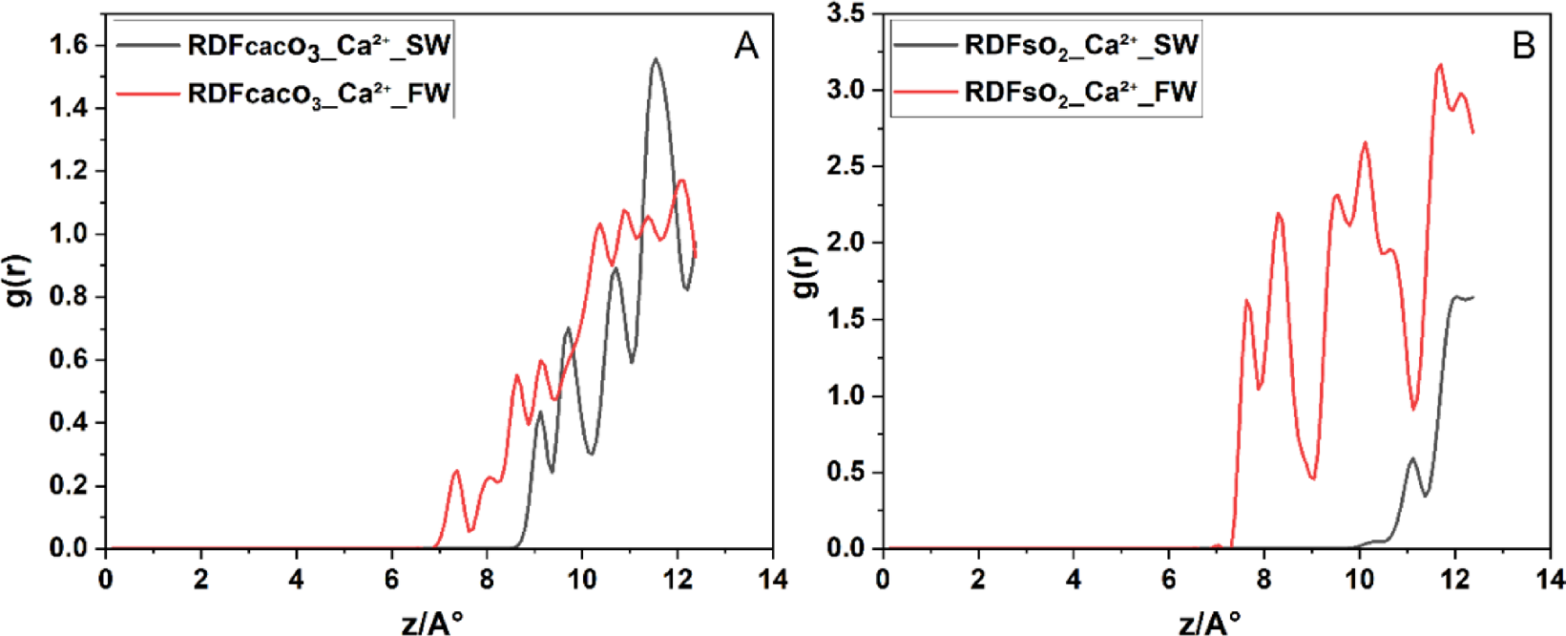
Radial distribution functions g(r) of Ca²⁺ relative to surface oxygens on (**A**) calcite and (**B**) quartz facets under (SW, black) and (FW, red) salinities.

In Fig. 9A, the RDF of CaC**o**_3_-**Ca**^2+^, Ca²⁺ ions in SW exhibit a weak interaction with the surface, with the first RDF peak appearing at ~ 7.5 Å and an intensity of 0.2. In FW, this peak shifts to g(r) ≈ 9 Å and increases to g(r) ≈ 0.6. These low-intensity peaks in both cases suggest that Ca²⁺ does not strongly bind to the calcite surface, resulting in a relatively diffuse and unstable EDL, with limited impact on wettility alteration under both brine types. In contrast, near the quartz surface, Ca²⁺ shows a much stronger interaction. In sw, the first peak occurs at g(r) ≈ 7.5 Å with a high intensity of g(r) ≈ 1.75, suggesting strong adsorption onto the negatively charged quartz surface. In FW the peak shifts to a longer distance ~ 11 Å, indicating more distant accumulation. This behavior confirms that Ca²⁺ ions preferentially interact with quartz, especially under SW conditions.

From a practical standpoint, these findings indicate that in quartz-dominated rocks, the presence of divalent cations like Ca²⁺ (especially in FW) can lead to EDL disruption and stronger charge inversion, which may drive wettability alteration toward oil-wet conditions. These insights are crucial when designing low-salinity waterflooding strategies or interpreting ion-specific effects in carbonate reservoirs.

**Fig. 10:**
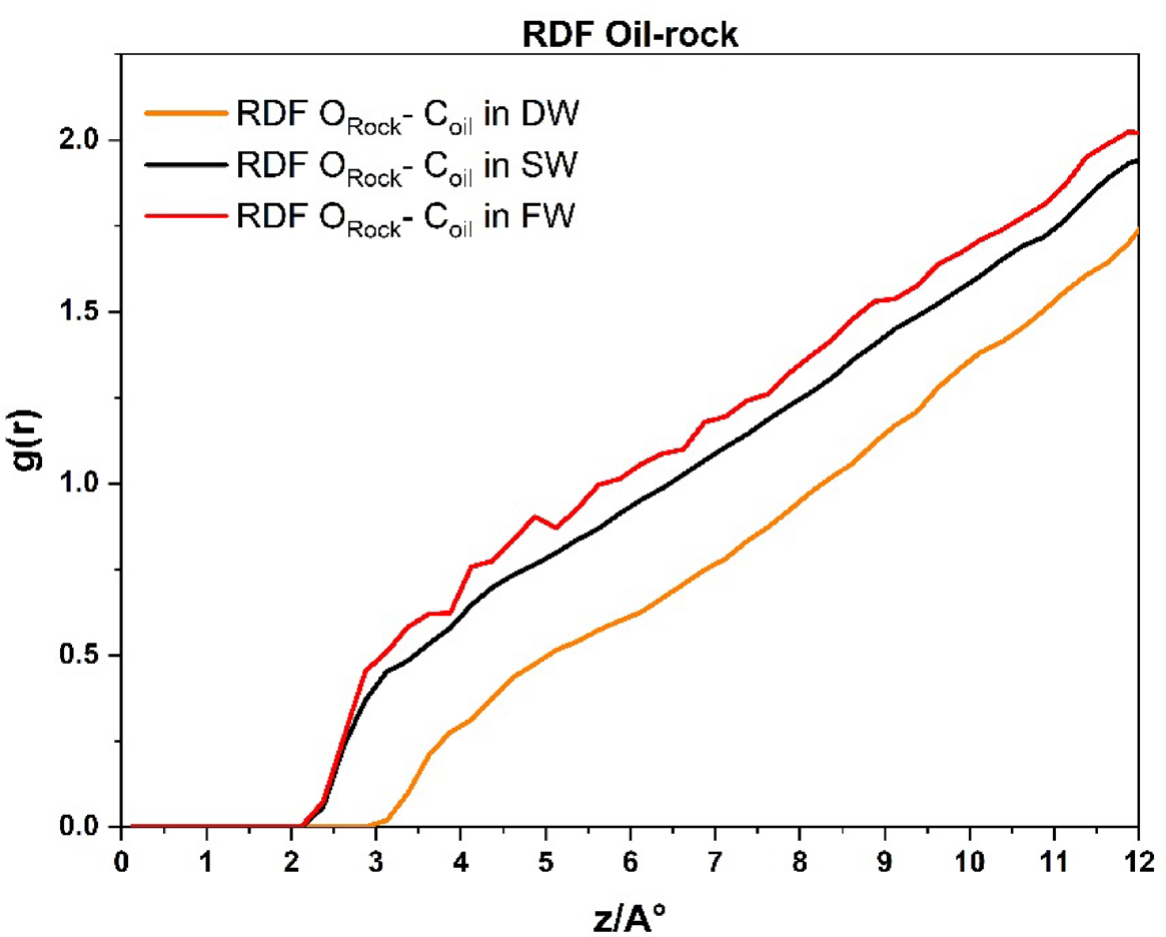
Radial distribution function plots between the carbon of the oil molecules and the oxygen of the carbonate reservoir rock in the cut-off radius of 12 Å for DW, SW, and FW *systems.*

Figure 10 presents the RDF between the carbon atoms of the oil molecules and the oxygen atoms of the carbonate rock surface, evaluated at a cut-off radius of 12 Å from the rock. This analysis aimed to assess the adhesion behavior of oil molecules to the rock surface under three different brine conditions. The results reveal that increasing salinity leads to a greater number of oil molecules interacting with the carbonate surface, which is consistent with previous studie^18,61^ the DW system exhibited the fewest interactions with the rock from the beginning. This suggests that DW injecting in carbonate rock exhibits the highest and most persistent degree of surface hydrophilicity.

**Fig. 11:**
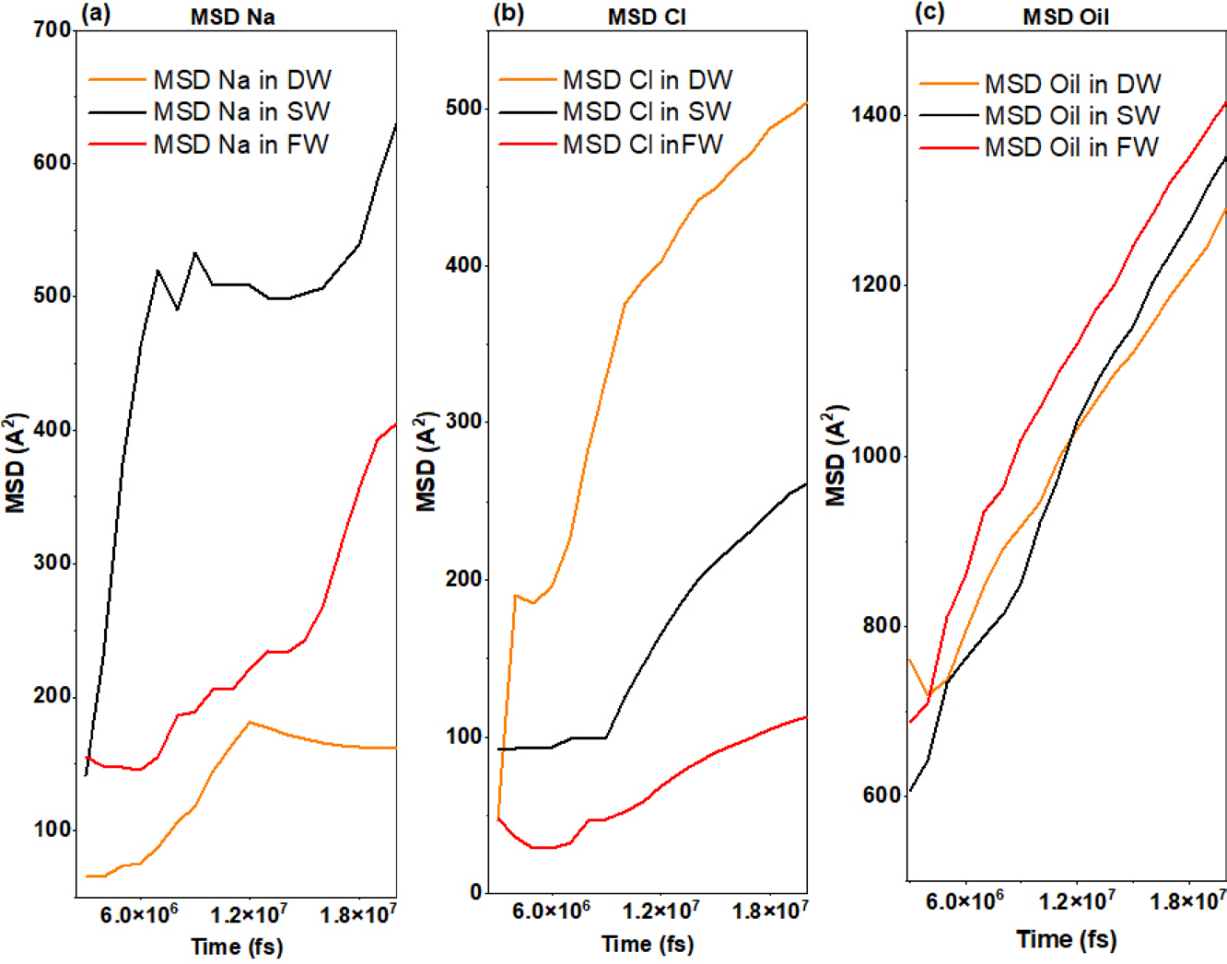
Mean Squared Displacement for each (**a**) Na + ions, (**b**) Cl- ions and (**c**) oil atoms for DW, SW, and FW systems.

MSD analysis in Fig. 11a was also used to check the movement of atoms during the simulation, which showed that Na + ions are the most mobile in the case of the SW system due to the most significant number of ions that interacted with the oxygen atoms of the rock surface at the beginning (approximately the first third) of the simulation. However, in the case of FW, due to the increase in the number of atoms in the porous medium, the movement of Na^+^ ions was reduced by half, which is due to the occupation of the oxygen atoms of the rock surface in a faster time frame and at the very beginning of the simulation, which is due to the increase in the number of sodium atoms. In the case of the DW system, its mobility was the least due to the stable distribution of the limited number of Na^+^ ions in the position of the oxygen atoms of the rock surface. Figure 11b, related to the MSD of Cl^−^ ions, provides that in all systems, Cl^−^ ions first show a small mobility, which is due to the adsorption of Cl^−^ into the quartz tissue; then, after the occupation of Silicon of quartz atoms by Cl^−^ ions, other Cl^−^ ions begin to move more freely (approximately in three-quarters of the simulation interval). An increase in salinity leads to a decrease in the movement of Cl^−^ ions because by interacting with each other, they create a mass of atoms of the same type, which play the role of a bridge between the oil and the reservoir rock, which, in turn, increases the adhesion of oil to the carbonate rock and reduces the EOR. The MSD diagram of the oil in Fig. 11c clearly shows that the decrease in salinity reduces the mobility of the oil and, as a result, reduces the adhesion of the oil to the carbonate rock surface and improves the EOR.

**Fig. 12:**
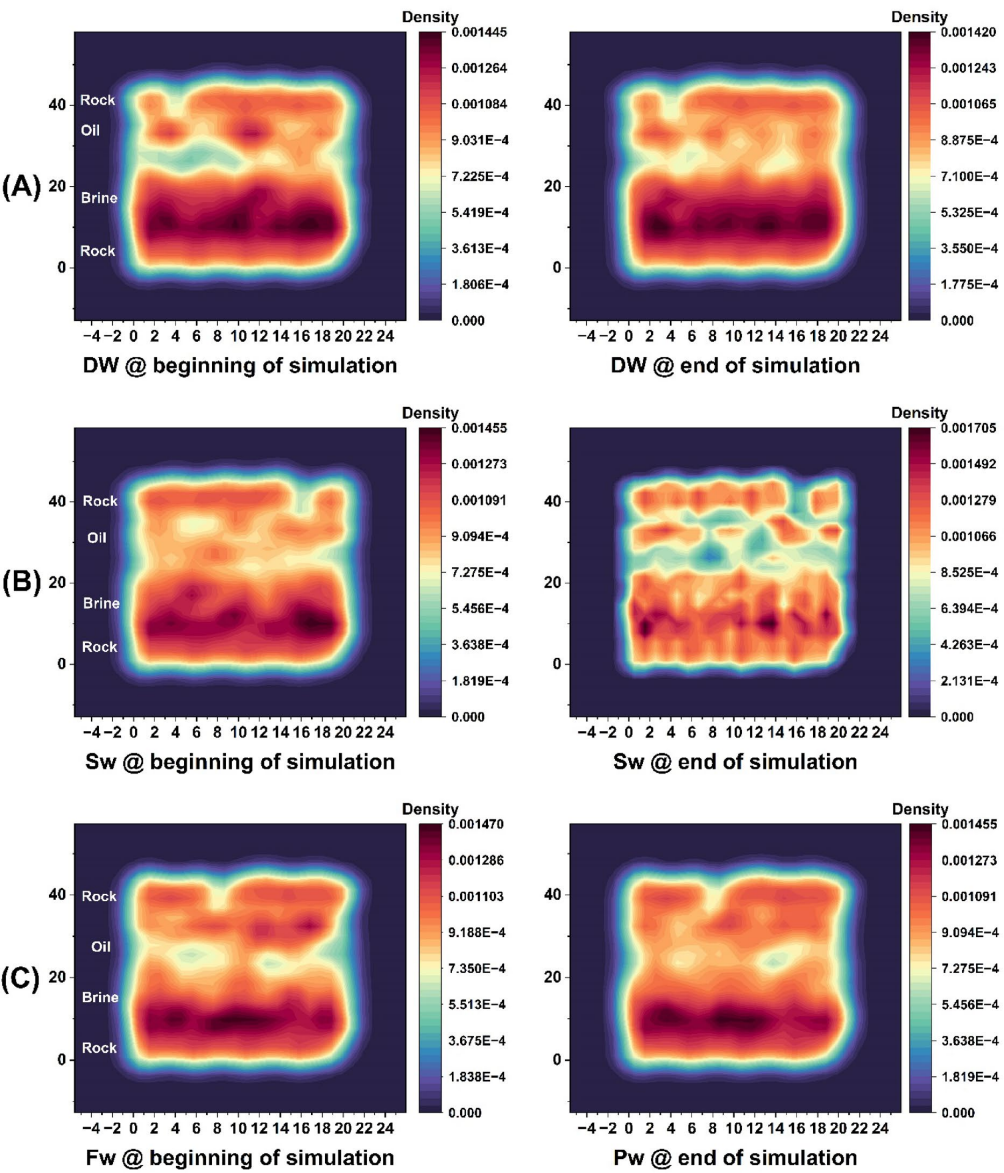
Two-dimensional density maps in the two-layer plane (ZY) for composite rock composed of calcite and quartz for three system states at the beginning of the simulation (left) and at the end of the simulation (right) from the production stage of the simulations.

Figure 12A, corresponding to the DW system, shows the upward migration of the oil phase away from the surface of the composite rock by the end of the simulation. This behavior is attributed to the disruption of ionic bridges between the oil and brine phases, which facilitates the detachment of oil from the rock surface and its movement toward production wells. Such detachment indicates a reduction in oil-wet, a condition favorable for EOR mechanism.

Figure 12B shows the SW system, with the morphology of oil and brine clearly distinguished at the final simulation stage. Here, ion clusters—particularly divalent cations (Ca²⁺-Mg²⁺) form bridging structures that interconnect oil masses with the carbonate surface. These cation-mediated bridges promote oil adhesion to the rock, which is unfavorable for EOR mechanism.

Similarly, Fig. 12C depicts the FW system, where abundant divalent ions in the high-salinity brine create strong ionic bridges at the oil–rock interface. This results in enhanced adhesion of oil to the carbonate reservoir rock and reduced oil mobility. The dominant role of Ca²⁺ and Mg²⁺ ions in governing electrostatic interactions and wettability alteration is thus clearly demonstrated across the different brine compositions. To provide further clarification, the initial and final atomic configurations for all systems are presented in figure.S9 of the Supplementary Information.

## Conclusion

This study reveals the critical role of salinity in influencing the thickness of the EDL in carbonate reservoir rocks. It also shows that the thinner EDL is caused by increased salinity, which results in stronger electrostatic interactions between Na⁺ and Cl⁻ ions. The thickness of the EDL has a clear impact on the wettability of the rock; the greater the thickness of the EDL promoting water-wet conditions, which are favourable for EOR. This study also shows the role of quartz in the carbonate reservoir rock, where the positive charge of silicon atoms of quartz attracts Cl⁻ ions and repels Na⁺ ions, disrupting the EDL, which in turn reduces the hydrophilicity of the surface of the rock. Furthermore, water molecules show a preference for interacting with calcite surfaces via hydrogen atoms, whereas both hydrogen and oxygen atoms in water exhibit similar tendencies to bond with quartz surfaces. Furthermore, increased brine ions in SW and FW systems cause the creation of ion bridges in the brine, creating a pathway for contact with the composite rock surface. This phenomenon strengthens the adhesion between oil and rock. These findings provide valuable insight into the complex fluid dynamics of underground reservoirs and have important implications for optimising EOR mechanisms.

## Supplementary Information

Below is the link to the electronic supplementary material.

Supplementary Material 1

## Data Availability

The data supporting the findings of this study have been deposited in Zenodo and are publicly available at: https://doi.org/10.5281/zenodo.15535266.

## Abbreviations

MDs: Molecular dynamics simulation
DW: Diluted water
SW: Sea water
FW: Formatin water
EDL: Electrical double layer
EOR: Enhanced oil recovery
LSWF: Low salinity water flooding
IFT: Interfacial tension
MIE: Multi-ion exchange
CVFF: Constant valence force field
NVT: Canonical ensemble (fix N, constant V and constant T)
RDF: Radial distribution function
MSD: Mean squared displacement
TIP3P: Transferable intermolecular potential 3-point
PPPM: Particle-particle-particle-mesh
VMD: Visual molecular dynamics software
