# Supplementary material for: Novel insights into electrical double layers in carbonate reservoirs under low-salinity water injection using molecular dynamics simulation
Source: Sci Rep. 2025 Aug 23;15:31061. doi: 10.1038/s41598-025-14647-z (PMC12375000; doi:10.1038/s41598-025-14647-z)
Supplement: Supplementary file 1 — Supplementary Material 1 [file 41598_2025_14647_MOESM1_ESM.docx]

**Electronic** **Supplementary Information**

**Novel Insights into Electrical Double Layers in Carbonate Reservoirs Under Low-Salinity Water Injection Using Molecular Dynamics Simulation**

Saifali Al-Musawi^1^, Fariborz Rashidia,^2,*^, Sepideh Amjad-Iranagh^3^

^1^ Department of Petroleum Engineering, Amirkabir University of Technology, (Tehran Polytechnic), Tehran, Iran

^2^ Department of Chemical Engineering, Amirkabir University of Technology, (Tehran Polytechnic), Tehran, Iran

^3^ Department of Materials and Metallurgical Engineering, Amirkabir University of Technology, (Tehran Polytechnic), Tehran, Iran

*Corresponding author: rashidi@aut.ac.ir

**Table S1**: . Reservoir fluid analysis.

| *Composition (mol % )* | | | | |  |  |
| --- | --- | --- | --- | --- | --- | --- |
| Component | Reservoir fluid | |  |  |  |  |
| N2 | 0.09 | |  |  |  |  |
| CO2 | 3.38 | |  |  |  |  |
| H2S | 0.30 | |  |  |  |  |
| CH4 | 65.64 | |  |  |  |  |
| C2H6 | 6.02 | |  |  |  |  |
| C3H8 | 3.09 | |  |  |  |  |
| iC4H10 | 0.43 | |  |  |  |  |
| nC4H10 | 1.08 | |  |  |  |  |
| iC5H12 | 0.58 | |  |  |  |  |
| nC5H12 | 0.70 | |  |  |  |  |
| Pseudo C6 | 2.38 | |  |  |  |  |
| Pseudo C7 | 2.03 | |  |  |  |  |
| Pseudo C8 | 2.17 | |  |  |  |  |
| Pseudo C9 | 1.86 | |  |  |  |  |
| Pseudo C10 | 1.62 | |  |  |  |  |
| Pseudo C11 | 1.46 | |  |  |  |  |
| C12+ | 7.20 | |  |  |  |  |
| Total | 100.00 | |  |  |  |  |
| *Molar Mass g/mole* | | | |  |  |  |
| Component | Reservoir fluid | |  |  |  |  |
| C7+ | 226.6 | |  |  |  |  |
| C12+ | 364.0 | |  |  |  |  |
| Average | 201.4 | 20.61 | | | | 56.1 |

**Table S2:** Molecular species and their respective counts included in the simulated oil sample.

| Molecule | Number of Molecules |
| --- | --- |
| CO₂ | 4 |
| Methane (CH₄) | 63 |
| Ethane (C₂H₆) | 6 |
| Propane (C₃H₈) | 4 |
| n-Butane (C₄H₁₀) | 2 |
| Hexane (C₆H₁₄) | 3 |
| Heptane (C₇H₁₆) | 2 |
| Octane (C₈H₁₈) | 2 |
| Nonane (C₉H₂₀) | 2 |
| Decane (C₁₀H₂₂) | 1 |
| Undecane (C₁₁H₂₄) | 2 |
| Dodecane (C₁₂H₂₆) | 7 |

**XRF and XRD Analyzes:**

Table S3: XRF analysis results of the laboratory core sample.

| Core sample | $842-3$ | L.O.I. | $\mathrm{CaO}$ | SiO_2_ |
| --- | --- | --- | --- | --- |
|  | (%) | $40.58$ | $51.703$ | $3.127$ |
| Na_2_O | $\mathrm{MgO}$ | $\mathrm{Al}_{2}O_{3}$ | $P_{2}O_{5}$ | $\mathrm{Sr}$ |
| $0.214$ | $0.664$ | $1.623$ | $0.015$ | $0.035$ |
| $SO_{3}$ | $\mathrm{Cl}$ | $K_{2}O$ | $\mathrm{Ti}O_{2}$ |  |
| $0.563$ | $0.107$ | $0.328$ | $0.076$ |  |
| $\mathrm{MnO}$ | $\mathrm{Fe}_{2}O_{3}$ | $\mathrm{Br}$ | $\mathrm{Zr}$ |  |
| $0.036$ | $0.92$ | $0.003$ | $0.006$ |  |

Table S3: provides a comprehensive list of the identified elements in the sample, along with their corresponding percentage compositions. The components CaO and SiO₂ exhibited the highest concentrations, with values of 51.703% and 3.12%, respectively. The other components, due to their relatively low concentrations, were excluded from the simulations to optimize computational resource allocation and enhance the overall accuracy of the results.


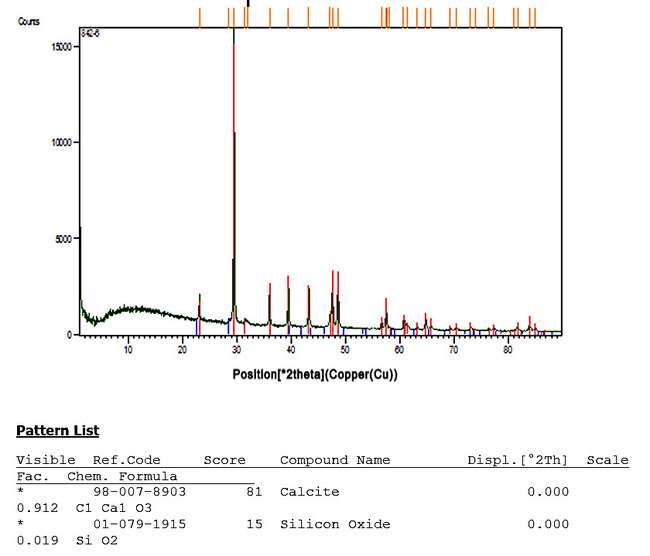


| Pattern List |  |  |  |  |  |
| --- | --- | --- | --- | --- | --- |
| Visible | Ref.Code | score | Compound Name | Displ. $\left[ {}^{\circ}2Th \right]$ | scale |
| Fac. | Chem. Formula |  |  |  |  |
| * | $98-007-8903$ | 81 | Calcite | 0.000 |  |
| 0.912 | C1 Ca1 o3 |  |  |  |  |
| * | $01-079-1915$ | 15 | Silicon oxide | 0.000 |  |
| 0.019 | Si02 |  |  |  |  |

**Figure. S1:** Graphs from the XRD analysis, as well as the reported results from the laboratory core sample.

The calculations performed from these two analyzes indicate that the structure of the composite reservoir rock is mainly of two types of calcite and quartz rocks with percentages of 87 and 13%, respectively.

## **System evaluation**

1. **Viscosity** **evaluation**

Numerous atomistic simulation methodologies for evaluating the shear viscosity of liquids have been developed. The GreenKubo relation, which is based on equilibrium (MD) simulations, is the most often utilized technique due to its simplicity. The GreenKubo method is used to determine the shear viscosity by integrating the pressure tensor autocorrelation function across time.

$$\eta=\frac{V}{k_{B}T}\int_{0}^{\infty} \left\langle P_{\alpha\beta}(t)\cdot P_{\alpha\beta}(0) \right\rangle dt Eq. (6)$$

where V is the volume of the system, kB denotes the Boltzmann constant, T signifies the temperature, Pαβ is Considered to be an indicator of the element αβ of the pressure tensor, and the angle bracket denotes the average of the ensemble.

After equilibration in the isothermal isobaric (NPT) ensemble, the systems were produced in the canonical (NVT) ensemble. Temperature and pressure were controlled using the NoseHoover thermostat and the extended Lagrangian method, respectively. The simulations were conducted at seven different pressures, as indicated in Table 4 which the values of oil viscosity in experimental and simulation conditions are presented. Reported for seven different pressures at 420 ° K temperature.

**Table S4:** Table includes the reported numbers of viscosities obtained in the experimental and computer simulations.

| pressure (psi) | Experimental viscosity (cs) | Simulation viscosity (cs) |
| --- | --- | --- |
| 7015 | 0.163 | 0.181 |
| 6215 | 0.150 | 0.142 |
| 6015 | 0.147 | 0.1449 |
| 5876 | 0.144 | 0.113 |
| 4015 | 0.238 | 0.213 |
| 2415 | 0.391 | 0.251 |
| 1615 | 0.475 | 0.214 |

***Figure S2***. *Oil viscosity graph in terms of pressure. Blue for laboratory viscosity and red for simulated viscosity.*

As illustrated in Fig. S2, at pressures below 3000 psi, the experimental and simulated viscosities are only slightly different; nevertheless, as pressure increases, a very excellent match is achieved between the experimental and simulated viscosities. It should be noted that all of the simulations in this research are performed under high pressure and temperature (field conditions).

1. **Contact angle measurement evaluation**

In order to evaluate the rock used in the simulation, the contact angle calculation method is used, which is as follows: First, for each rock model, a separate system was designed to calculate the contact angle with the assumption of water fluid, as shown in Fig.S3 and Fig.S4 of the designed system. For both systems, the volume is assumed to be 2*2*2 nm for water box placed on quartz surfaces and calcite surfaces, respectively. It was decided to start with the equilibration of the systems in the canonical (NVT) ensemble by the temperature 300.0 K for 100000 fs.


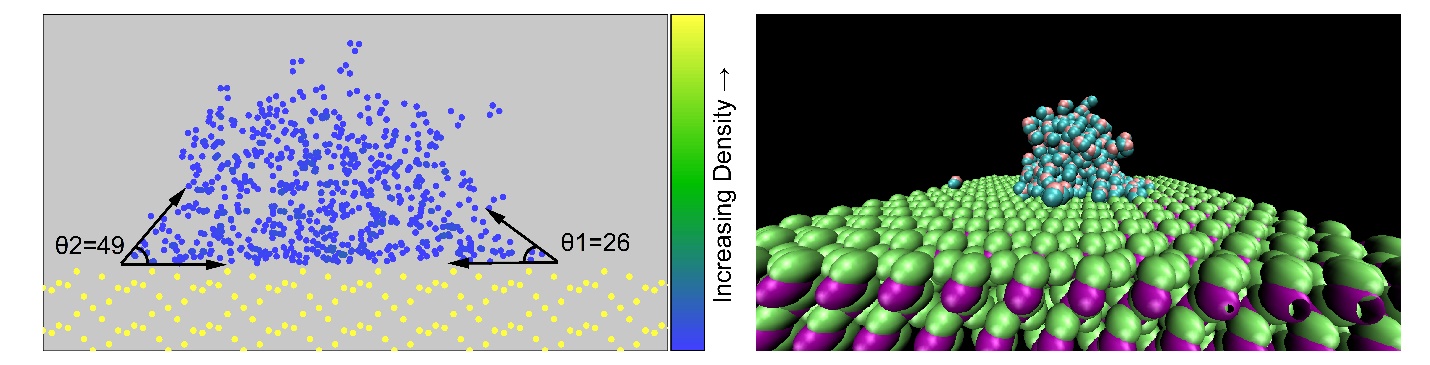


***Figure S3***. *On the above side of the two-dimensional image of the water drop density profile on the Sio2 surface, which was calculated from two directions of contact angle, and at the down side, the three-dimensional image of the water-quartz system is expressed.*

In the simulation of the quartz surface, contact angle values of 26 and 49 degrees were obtained. These values agree with those obtained from the work Deng, Y et al. 2018^44^.


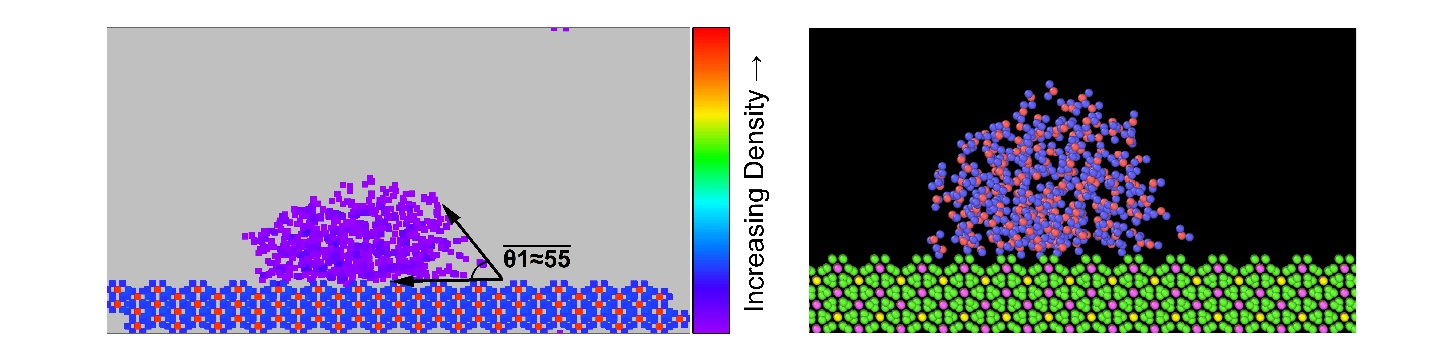


***Figure S4*.** *Two-dimensional image of the water droplet density profile on the CaCO_3_ surface in the left. The three-dimensional image of the water-calcite system is at the right, where the contact angle is* *θ1**≈55.*

To simulate the water-calcite contact surface, an angle of 55 degrees was acquired, as illustrated in Fig. S4. This is in excellent agreement with the work done by Kowalczuk, P. B. et al^40^, who obtained a contact angle of *≈* 60 degrees in their work.


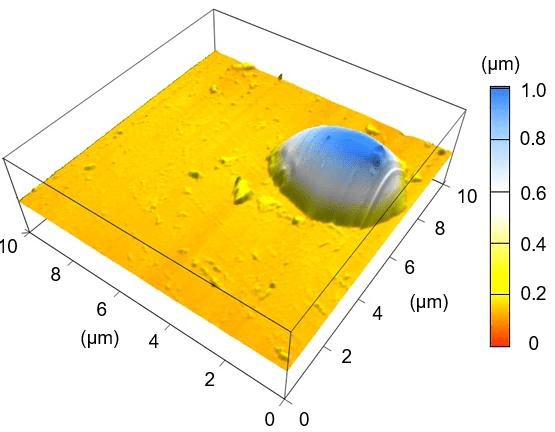

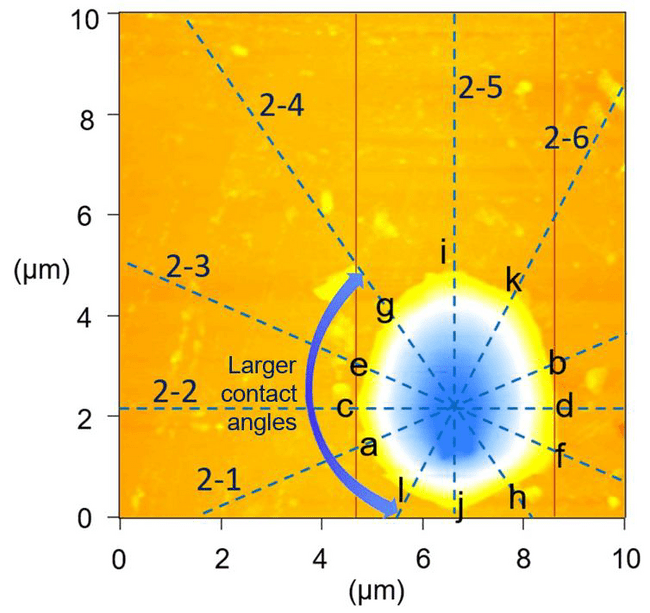


|  | Line 2-1  a b | Line 2-2  c d | Line 2-3  e f | Line 2-4  g h | Line 2-5  i j | Line 2-6  k l |
| --- | --- | --- | --- | --- | --- | --- |
| Contact Anglesº | 44.9 39.3 | 42.7 33.9 | 45.2 27.8 | 47.1 42.8 | 38.1 35.5 | 35.8 48.9 |

***Figure S5****. A typical AFM result of a water droplet (~5 μm in diameter) on the quartz 1 surface was studied. (a) 3-D topography of the droplet; (b) Top view of the droplet. Dashed lines from 2-1 to 2-6, crossing the water. Droplet along which the 2-D configurations are acquired for contact angle measurement. Additional, the contact angles along the contact line of the water droplet on quartz surface^44^.*

**Potential Models and Force Fields Parameters**

By employment of a Consistent Valence Force Field (CVFF), a general force field for organic molecules, was utilized to identify the parameters of organic species. CVFF allowing the integration of properties from both force fields in hybrid organic-inorganic systems. CVFF force fields have been widely utilized to model the interactions of rock textures and organic species. The CVFF force field was employed to explain oil contact with Na+, Cl−, K+, and Ca^+2^ ions interaction parameters. This set of parameters has been proved to be compatible with CaCO_3_ and Sio_2_. A geometric mixing rule was used to model the interactions between species. All the simulation was conducted using the real unit system.

$\begin{aligned} A_{\mathrm{ij}}=\sqrt{A_{i}\times A_{j}} \\ B_{\mathrm{ij}}=\sqrt{B_{i}\times B_{j}} \end{aligned}$ $Eq.$ (1)

where $A$ and $B$ are the Lennard-Jones ($\mathrm{LJ}$) potential's (eq. 1) parameters. The van der Waals and electronic contributions are described by the LJ 12-6 and the Coulomb potential, respectively.

$$E_{LJ+Coul}=\left[ \frac{A}{r_{\mathrm{ij}}^{12}}-\frac{B}{r_{\mathrm{ij}}^{6}} \right]+\frac{e^{2}}{4\pi\varepsilon_{0}}\cdot\frac{q_{i}q_{j}}{r_{\mathrm{ij}}} Lennard-Jones Potential Equation Eq.(2)$$

where $r_{\mathrm{ij}}$ represents the distance between atoms $i$and $j$, $q$ represents the atomic charge, and $\varepsilon_{0}=8.85418782\times{10}^{-13}F\cdot\mathrm{nm}^{-1}$ represents the vacuum permittivity. Table S5 provides a comprehensive presentation of the LJ 12-6 and charge parameter of each simulation atoms. Covalent bond energies, bond angles, and dihedral angles are described by

$$\begin{aligned} &E_{\text{bond }}=\sum_{i} K_{i}^{r}\left( r_{i}-r_{i0} \right)^{2} Eq.(3) \\ &E_{\text{angle }}=\sum_{i} K_{i}^{\theta}\left( \theta_{i}-\theta_{i0} \right)^{2} Eq. (4) \\ &E_{\text{dihedral }}=\frac{1}{2}\sum_{i} \sum_{n=1}^{4} V_{n.i}\left[ 1+(-1)^{n+1}cos\left( n\varphi_{i} \right) \right] Eq. (5) \end{aligned}$$

In which the equilibrium bond lengths and covalent angles are, respectively, $r_{i0}$ and $\theta_{i0}$, the $K$ values represent the stiffness of each, and $\varphi_{i}$ is the dihedral angle.

# **Minimization of energy**

Because the drawn chemical structures are not energetically beneficial, minimizing energy is necessary. Molecular potential energy encompasses components such as stretching, bending, and torsion. Molecular modeling (MD) allows for the most stable conformation. Hence, Style CG is the Polak-Ribiere version of the conjugate gradient (CG) algorithm is used. In order to reach 1e-8 stopping tolerance energy and 1e-8 stopping tolerance for force as minimized parameters. The minimized energy and equilibrium both pressure and temperature are performed for the simulation. Also, the system is relaxed for 0.5 ps using the isothermal-isobaric ensemble (NPT) where a 426-atmosphere thermostat is used. With a 0.1 limit where the Langevin thermostat is employed.

**Figure S6**. Diagram of equilibration pressure after 0.5 ns under NPT ensemble.

Fig. S6 for pressure minimization shows that the system reaches the desired compression balance of 426 atmospheres after passing the 0.5 ns time. After that, the simulation is performed for 0.5 ns using the microcanonical ensemble (NVE) to achieve a pressure balance of 296 K.

**Figure S7.** Diagram of equilibration temperature after 0.5 ns under NVT ensemble.

Fig. S7 clearly shows the equilibrium temperature function of 296 clones designed in the system. Simulation details Atomistic simulation is performed using the lammps open-source software, as a forcefield The organic species' forcefield parameters (FFP) were derived from the CVFF, a general force field for organic molecules. CVFF force field have been used extensively to model the interactions of clay minerals.

**Table S5:** Force-field parameters for system atoms.

|  | A12 | B6 | charge | element |
| --- | --- | --- | --- | --- |
| 1 | 0.121235 | 2.812953 | 1.668 | ca+ |
| 2 | 0.121235 | 2.812953 | - | ca^up^ |
| 3 | 0.148 | 3.617049 | 0.999 | c- |
| 4 | 0.228 | 2.859785 | -0.889 | o- |
| 5 | 0.148 | 3.617049 | -0.550 | c-+ |
| 6 | 0.228 | 2.859785 | - | o-^up^ |
| 7 | 0.228 | 2.859785 | -0.550 | oz |
| 8 | 0.040018 | 4.053434 | 1.10000 | sz |
| 9 | 0.228 | 2.859785 | - | oz^up^ |
| 10 | 0.040018 | 4.053434 | - | sz^up^ |
| 11 | 0.228 | 2.859785 | -0.150 | o |
| 12 | 0.16 | 3.474505 | -0.400 | c |
| 13 | 0.038 | 2.449971 | 0.100 | h |
| 14 | 0.039 | 3.875409 | -0.300 | c3 |
| 15 | 0.039 | 3.875409 | -0.200 | c2 |
| 16 | 0.155416 | 3.16552 | -0.820 | o* |
| 17 | 0 | 0 | 0.410 | h* |
| 18 | 1.607143 | 1.897439 | 1 | Na |
| 19 | 0.121235 | 2.812953 | 2 | ca^brin^ |
| 20 | 0.107 | 4.446297 | -1 | Cl |
| 21 | 0.040018 | 4.053434 | 1 | mg |

# **Equilibration protocol**

The procedures below, which are also shown in Fig. S8, were used to establish the fluid's thermodynamic state inside the calcite slit:

(1) The upper textural rock was considered as a rigid body, and the bottom textural rock slab was kept immobile. In addition, the top rock atoms were counted as zero charge and served only as the simulation's piston to keep them from contributing to the energy calculations. To reach 420 k, the NVT ensemble was applied for 0.5 ns to oil molecules and brine solution. Variations in potential energy and the top rock slab's elevation over time are depicted in Fig. S8 (2).

(2) After setting the temperature, the brine solution was compressed by uniformly exerting a force equivalent to 420 atmospheres on the upper textural rock slab. This action was continued for 0.5 ns at the NVT ensemble, as shown in Fig. S8 (3).

(3) Finally, when the oil molecules and brine solution reached the temperature and pressure of the actual reservoir conditions being studied, the upper texture rock was fixed. Under these conditions, the production simulation was run for 20 ns, as illustrated in Fig. S8 (4).


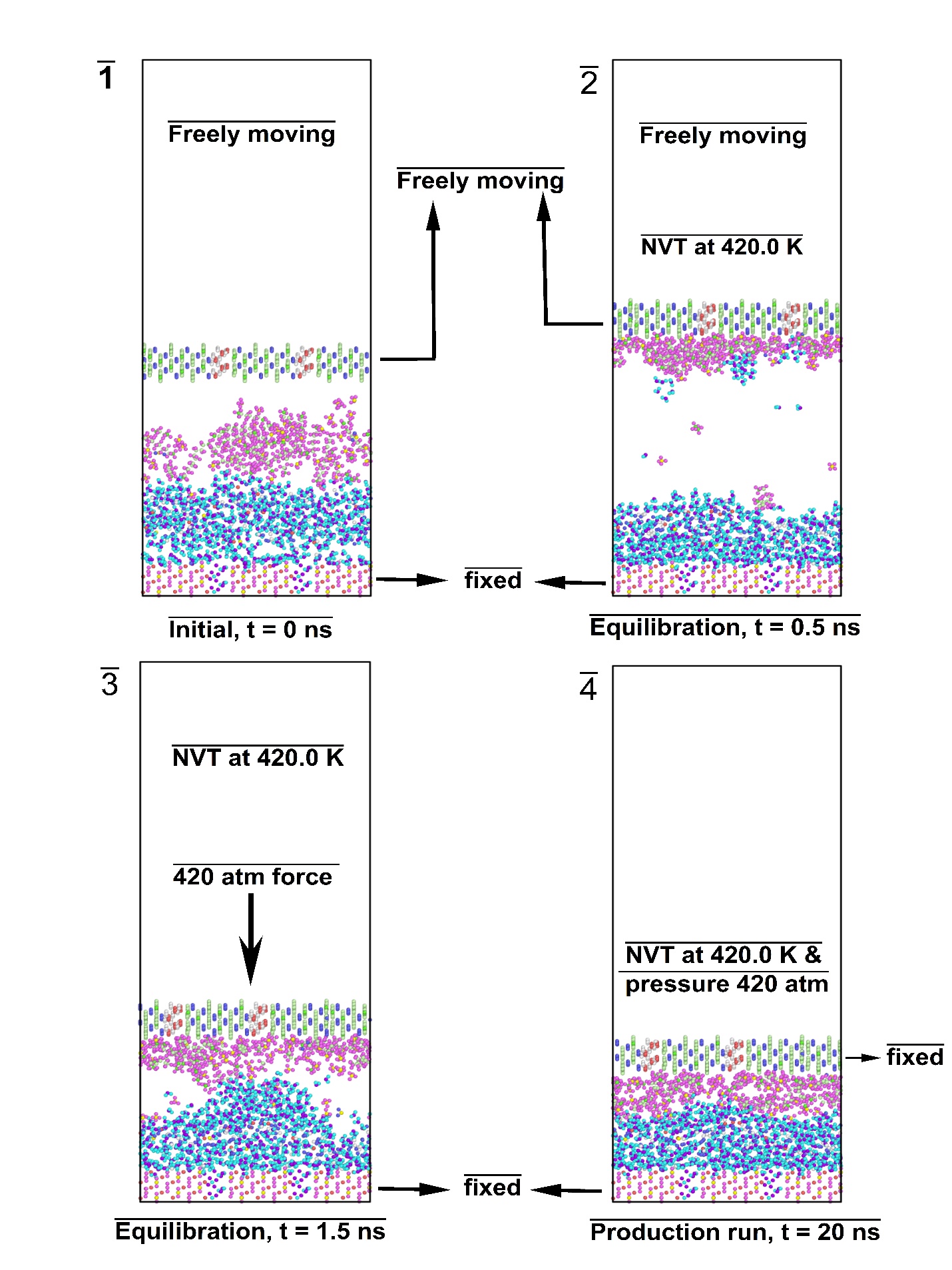


**Figure S8.** Simulation steps performed to reach the thermodynamic state of the real reservoir conditions.


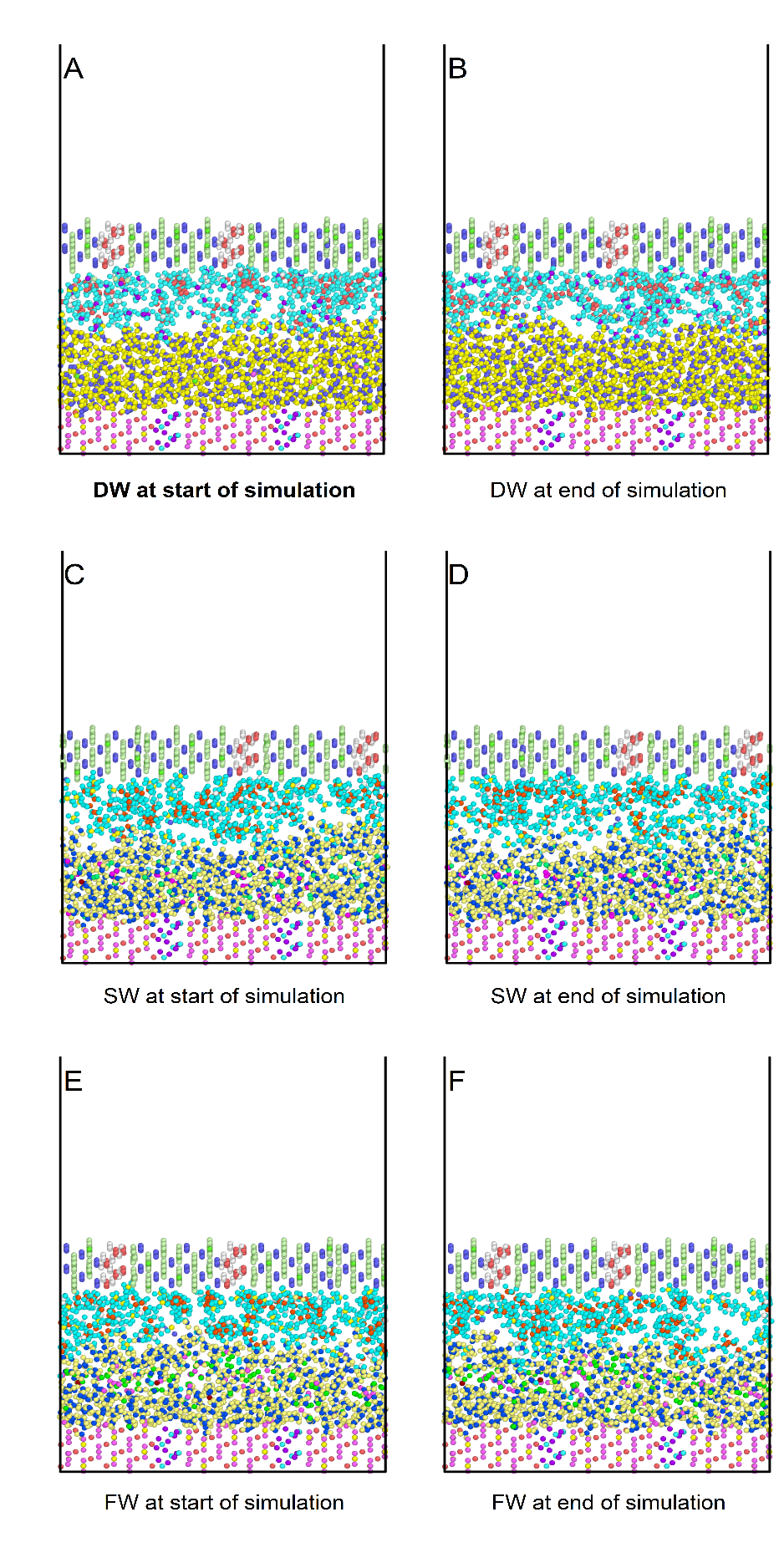


**Figure S9**: Initial (t = 0 ns) and final (t = 20 ns) md snapshots configurations of oil–brine interfacial films on a mixed calcite–quartz surface.
